# Profiling the serum proteome during *Schistosoma mansoni* infection in the BALB/c mice: A focus on the altered lipid metabolism as a key modulator of host-parasite interactions

**DOI:** 10.3389/fimmu.2022.955049

**Published:** 2022-08-31

**Authors:** Gustavo Gonçalves-Silva, Lara Geralda Magela dos Santos Vieira, Miguel Cosenza-Contreras, Ana Flávia Pinho Souza, Daniela Caldeira Costa, Wiliam Castro-Borges

**Affiliations:** ^1^ Programa de Pós-Graduação em Ciências Biológicas, Universidade Federal de Ouro Preto, Ouro Preto, Brazil; ^2^ Faculty of Biology, University of Freiburg/Institute for Surgical Pathology, University Medical Center, Freiburg, Germany; ^3^ Departamento de Ciências Biológicas, Instituto de Ciências Exatas e Biológicas Universidade Federal de Ouro Preto, Ouro Preto, Brazil

**Keywords:** schistosomiasis, serum proteome, lipid metabolism, host-parasite interaction, mass spectrometry

## Abstract

Schistosomiasis represents a condition in which every aspect of the disease, starting from skin invasion of the cercariae to egg laying by adult worms, incites a tissue response from the vertebrate host. This response, whether acute or chronic, leads to the appearance of reporter molecules of tissue injury in bodily fluids that could be surveyed as markers for disease diagnosis, status and prognosis. In this scenario, the serum proteome associated with a schistosome infection remains poorly explored; particularly by the use of high-throughput mass spectrometric instrumentation. In this study, we aimed to comparatively examine the serum proteome of control versus infected BALB/c mice, spanning the interval between the onset of egg laying and the peak of the acute phase of infection. Compositional analysis of the sera, using one dimensional reversed-phase fractionation of tryptic peptides coupled to mass spectrometry, allowed identification of 453 constituents. Among these, over 30% (143 molecules) were differentially present comparing sera from infected and non-infected mice, as revealed by quantitative label-free shotgun approach. The majority of proteins exhibiting altered levels was categorised as belonging to immune response (acute phase-related proteins) followed by those linked to lipid transport and metabolism. Inspection of the lipid profile from control and infected individuals demonstrated more pronounced and significant alterations in triglycerides, VLDL and HDL fractions (p<0,001), attesting for a disturbance in circulating lipid molecules, and suggesting a key role in host-parasite interactions. Our findings provide a global view of the serum proteome in the context of experimental schistosomiasis during the acute phase of infection. It contributes by listing key molecules that could be monitored to inform on the associated inflammatory disease status. We hope it will shed light into uncovered aspects of the *Schistosoma mansoni* parasitism in the vertebrate host, particularly those related to modulation of the lipid metabolism mediating immune responses.

## Introduction

Schistosomiasis is a neglected tropical disease affecting millions of people worldwide (1–3). Its clinical manifestations promote in most cases irreversible tissue damage to the host, resulting in a high morbidity disease (4). *Schistosoma mansoni* adult worms dwell for years within the host vasculature, where they interact with circulating host molecules and utilise the nutritional rich constituents of sera to sustain parasitism. Molecular investigations have provided a deep understanding of parasite´s biology (5–7), however much remains to be elucidated on the mechanisms operating in this complex host-parasite interaction. Understanding host responses to schistosomes could reveal biomarkers of active infection and allow for better monitoring on the inflammatory status as a manner to alleviate the long term chronic tissue injuries.

Proteomic investigations aimed at elucidating *S. mansoni*-host interactions have been applied to the liver (8–10) and spleen (11) soluble proteomes in the murine model of the disease. In these occasions, significant changes in protein expression were observed, but how these are triggered is poorly understood. Therefore, analysis of the serum proteome in the context of schistosomiasis could provide the basis to comprehend the metabolic reprogramming operating at target tissues. Recent studies have investigated the serum proteome during *S. japonicum* infection in animal models of the disease and markers of infection have been proposed (12, 13). To our knowledge, we are the first to investigate the serum proteome in the context of *S. mansoni* infection, in the murine model, with a particular focus on proteins related to lipid metabolism.

Here we employed quantitative label-free shotgun proteomic analyses of the serum proteome collected at the 5^th^ and 7^th^ weeks post infection from *S. mansoni* infected BALB/c mice. Compositional analysis of the undepleted sera allowed identification of over 400 constituents, over 30% of which were differentially abundant comparing control and infected individuals. Distinct patterns of protein expression were seen at these two time points, possibly as a response to specific demands related to the onset of egg laying by adult parasites and granuloma formation in the liver. Our findings provide a repertoire of differentially abundant serum constituents in the context of *S. mansoni* infection and uncover key molecules proposed to modulate lipid metabolism during disease establishment.

## Materials and methods

### Ethics statement

All experiments involving animals were conducted in accordance with the Brazilian Federal Legislation (Arouca’s Law number 11,794). The routine methods for maintenance of the *S. mansoni* life cycle and the experimental procedures involving mice were reviewed and approved by the local Ethics Committee on Animal Experimentation ‘Comissão de Ética no Uso de Animais’ (CEUA), Universidade Federal de Ouro Preto (UFOP), and received the protocol numbers 2017/35 and 2017/34, respectively.

### Infection model and experimental design

Twenty-three male and female BALB/c mice aged 35 days were anaesthetised using a combination of ketamine hydrochloride (8 mg/kg) and xylazine (4 mg/kg) administered intraperitoneally. For infected groups, mice were exposed to active penetration by *S. mansoni* cercariae (LE strain) through tail immersion (250 cercariae/animal). The control group was established with 23 non-infected individuals of the same age. After 5^th^ and 7^th^ weeks post-infection, both infected and control mice were weighed and sacrificed using anaesthetic overdose. Spleens were collected and weighed to measure the spleen-to-body weight ratio Supplementary **Figure 1**. Serum samples were collected and their protein content measured using the BCA protein assay kit (Thermo Scientific, Cramlington, UK). Samples were kept frozen under -20°C and used for the analyses within a maximum of 60 days after collection from animals. The number of samples varied depending on the type of analysis. A total of 40 sera (n = 10 per group) proved suitable (did not display hemolysis and quantity was not limiting) for analysis of the lipid profile. For proteomic analysis sera from three animals from each group were pooled and this constituted one biological replicate per group. A second biological replicate was prepared by pooling three other sera from each group. Compositional and quantitative proteomic data were obtained from 6 independent LC-MS/MS runs.

### In-solution digestion

Briefly, 30 µg protein representing pooled sera from control or 5^th^ and 7^th^ post-infection groups were incubated with RapiGest (Waters, UK), to a final concentration of 1% (w/v) and maintained at 80°C for 10 min. Then, proteins were reduced using dithiothreitol (Sigma Aldrich, St. Louis, MO, USA) to a final concentration of 3,3 mM at 60°C for 10 min. Alkylation was performed at room temperature in the dark, using iodoacetamide (GE Healthcare, Little Chalfont, Buckinghamshire, UK) to a final concentration of 9,4 mM for 30 min. The digestion was performed using sequencing grade trypsin (Promega, Madison, WI, USA) at a ratio of 50:1 (protein/trypsin) during incubation at 37°C for 16 h. To precipitate RapiGest and stop the digestion step, trifluoroacetic acid was added to a final concentration of 0.5% v/v. Afterwards, a centrifugation step was performed for 15 min at 7°C at 20,000 x g (Mikro 200R, Hettich Zentrifugen, Tuttlingen, DEU). The clarified supernatant containing the peptides was then collected and submitted to UHPLC-MS/MS analyses.

### Liquid chromatography-mass spectrometry

After the digestion step, approximately 400 ng of peptide samples were injected into the liquid chromatography platform (UHPLC UltiMate R 3000, Dionex, San Jose, USA). Peptides were loaded onto an Acclaim PepMap100 C18 Nano-Trap column (100µm i.d. × 2 cm, 5 µm, 100 Å; Thermo Scientific, Waltham, MA, USA) and washed for 3 min in 3,8% acetonitrile/0.1% Trifluoroacetic acid solution (ACN, HPLC grade, USE), at a flow rate of 5 µL/min. Then, peptides were directed to a reverse phase chromatography using an Acclaim PepMap100 C18 RSLC column (75µm i.d. × 15 cm, 2 µm, 100 Å; Thermo Scientific), in tandem with the trap column on a constant flow rate of 300 nL/min. A multistep gradient was performed, using solvents A (0.1% formic acid, HPLC grade, JTBaker, Mexico) and B (80% ACN, 0.1% formic acid) and applied as follows: a conditioning step with 3,8% of B during 3 min, followed by a ramp from 3,8 to 30% B over 120 min, and subsequently, 30–55% B to 150 min. Then a final ramp with 99% B to 162 min, followed by a reconditioning step with 3,8% B until 180 min. The spectral data was acquired using a Q-Exactive mass spectrometer (Thermo Scientific, Bremen, Germany) operating at full-scan/MS2 mode. The nanospray flex ion source (Thermo Scientific) was set at 3,8 kV on positive mode, with a capillary temperature of 250°C and S-lens levels set to 55. The Data Dependent Acquisition method (DDA) was performed in the top 12 ions with charge states between +2 and +4 from a 1,2 *m/z* window. Survey scans were tuned for a resolution of 70,000 with a mass range between 300 and 2,000 *m/z*, and a AGC target of 1e^6^ ions in up to 120 ms. For MS/MS scans, selected ions were fragmented by Higher Energy Collision Dissociation (HCD) with a stepped Normalized Collisional Energy (NCE) of 28–30. After the dissociation, the fragmentation spectra were acquired using a resolution of 17,500 performing a maximum injection time of 60 ms with a dynamic exclusion time of 40 s and a target value of 5e^5^ (minimum AGC target 6.25e^3^).

### Analysis of proteomic data

The spectral data obtained from the UHPLC-MS/MS platform was directed to protein identity search using PEAKS Studio v8,5 (Bioinformatics Solutions Inc.). Searching parameters included: Enzyme: trypsin; maximum of 2 missed cleavage sites; fixed post-translational modifications: cysteine carbamidomethylation (+57,0214 Da) and methionine oxidation (+15,9949 Da) as variable modifications, maximum 3 post-translational modifications per peptide; time window: 4 minutes; mass error tolerance of 10 ppm for precursor ions and 0,1 Da for product ion fragmentation.

The database search was performed with a False Discovery Rate (FDR) of 1%, a protein significance of 13 (p-value ≤0.05) considering only proteins identified with at least one unique peptide. The *Mus musculus* proteome database was downloaded from the UNIPROT website, containing 55,398 protein sequences. Quantitative label-free analysis was performed under fold-change ≥ 2, protein significance of 13 (p-value ≤0,05) using the PEAKSQ significance method, considering proteins identified with at least 1 unique peptide. Cytoscape Software v. 3.9.1 (available at https://cytoscape.org/), underpinned by STRING query tool, was used to construct a protein interaction network for differentially abundant serum constituents and retrieve the top 10 most significant enriched molecular functions they are linked to.

### Lipidogram analyses

Lipidogram analyses were performed through measurement of Total Cholesterol, HDL Cholesterol and Triglycerides using colorimetric detection methods (BioClin-Quibasa kits, Belo Horizonte, MG, Brazil) using a Calm SBA2000 equipment (Medsystem). VLDL cholesterol levels were estimated by dividing triglyceride levels by 5, as specified in the literature (14). The atherogenic fraction was calculated by subtracting Total Cholesterol levels from HDL cholesterol. Sera from ten animals per group (control at 5^th^ and 7^th^ weeks post-infection and respective infected groups) were used for these analyses.

### Statistical analysis

Statistical analysis of data ​​obtained from body weights, spleen weights, and lipidogram parameters were evaluated for significant statistical differences (p-value ≤0,05), using the GraphPad Prism v8,0 program (San Diego, California, USA). The Kolmogorov-Smirnov and Shapiro-Wilk tests were applied to evaluate data normality. Then, the t-test or Mann-Whitney test were applied according to parametric or non-parametric distribution. All values were expressed as the mean ± standard deviation. For principal component analysis, the ClustVis (15) platform was used to perform the multivariate analysis.

## Results

### Assessing disease establishment during experimental schistosomiasis

In this investigation we first seek to evaluate disease progression in the BALB/c model of experimental schistosomiasis aiming to monitor the homogeneity of serum samples for downstream analysis from control and infected individuals. As shown in **Supplementary Figure 1A** the spleen from infected animals exhibited significant increase in size at 5^th^ and 7^th^ post infection, whilst for non-infected animals it remained unchanged. In addition, the observed splenomegaly throughout the infection period is better monitored by measuring the spleen-to-body ratio (9). As seen in **Supplementary Figures 1B**, **C** for both investigated periods, infected animals displayed a marked increase in this ratio compared to control animals, attesting for the successful establishment of murine schistosomiasis. As for the spleen, liver from infected animals were visually enlarged in both periods with increased granuloma formation, particularly at the later time point.

### The compositional analysis of undepleted sera in the context of murine schistosomiasis

**Figure 1:**
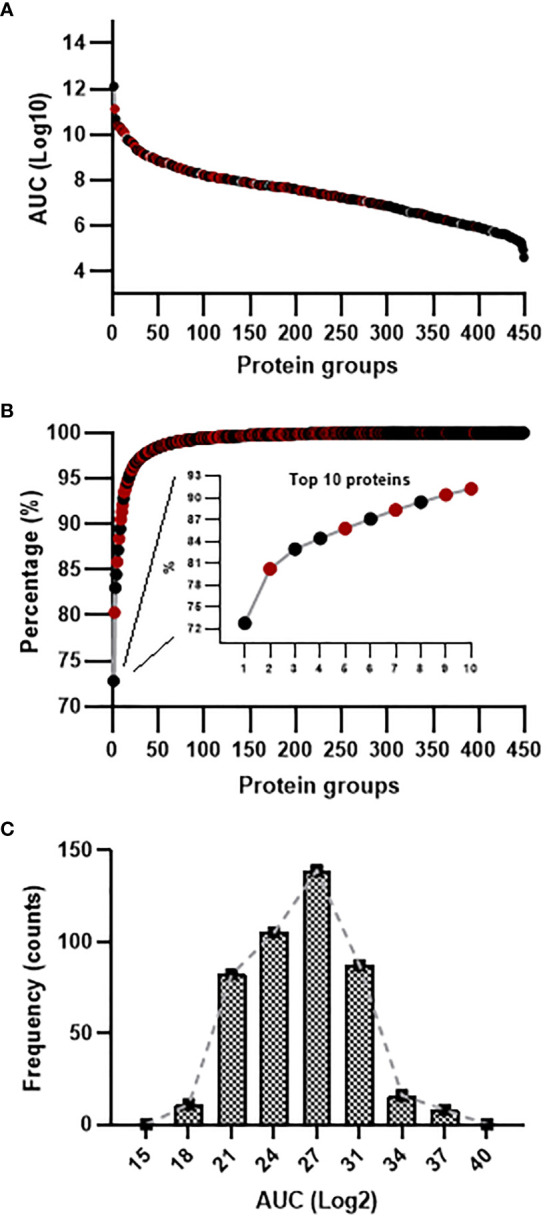
Compositional analysis of undepleted serum proteome during *S. mansoni* infection in the BALB/c mice. **(A)** Dynamic range of identified proteins encompassed five orders of magnitude difference between the most and least abundant serum constituent, as judged by Area Under Curve (AUC). **(B)** Cumulative abundance plot revealed 10 molecules contributing to 91% of the total ion signal. **(C)** Frequency histogram for protein abundance in the samples.

In order to evaluate the depth of proteome coverage through mass spectrometric analysis of undepleted murine sera, we first combined the spectral data obtained from uninfected and infected animals to interrogate the available *Mus musculus* proteome database. The accumulated 35,555 Peptide-Spectrum Matches resulted in 3450 confidently assigned peptide sequences yielding 453 distinct protein groups (**Supplementary Table 1**). Inspection of the total accumulated area for the top and least abundant constituents revealed five orders of magnitude difference, attesting for the identification of both high, medium and low abundant serum components (**Figure 1A**). Approximately 90% of cumulative abundance was accounted for 10 protein identities, the remaining 10% comprising the additional 443 molecules (**Figure 1B**). The top 10 most abundant proteins were mainly associated with transport, immune function and acute phase response. Serum Albumin was the most abundant component (72.7% of the detected ion signal), followed by Serotransferrin (7.5%) and Pregnancy zone protein (2.9%). Additional seven constituents in the top 10 list (Apolipoprotein A-I, Hemopexin, Immunoglobulin heavy constant mu, Complement C3, Immunoglobulin kappa constant, Alpha-2-HS-glycoprotein and Fibrinogen beta chain) collectively contributed 8,3% to the cumulative ion abundance. The vast majority of identified proteins contributed 8,7% to the total ion signal. This variation in the dynamic range of protein expression was confirmed by analysis of the frequency histogram for protein abundance, which revealed a non-Gaussian distribution (p<0,0001), **Figure 1C**. Next, we performed a comparative label-free quantitative analysis on the mass spectral data to detect differentially abundant serum molecules in the acute phase of murine schistosomiasis.

### The host serum proteome is pronouncedly altered during the acute phase of experimental schistosomiasis

**Figure 2:**
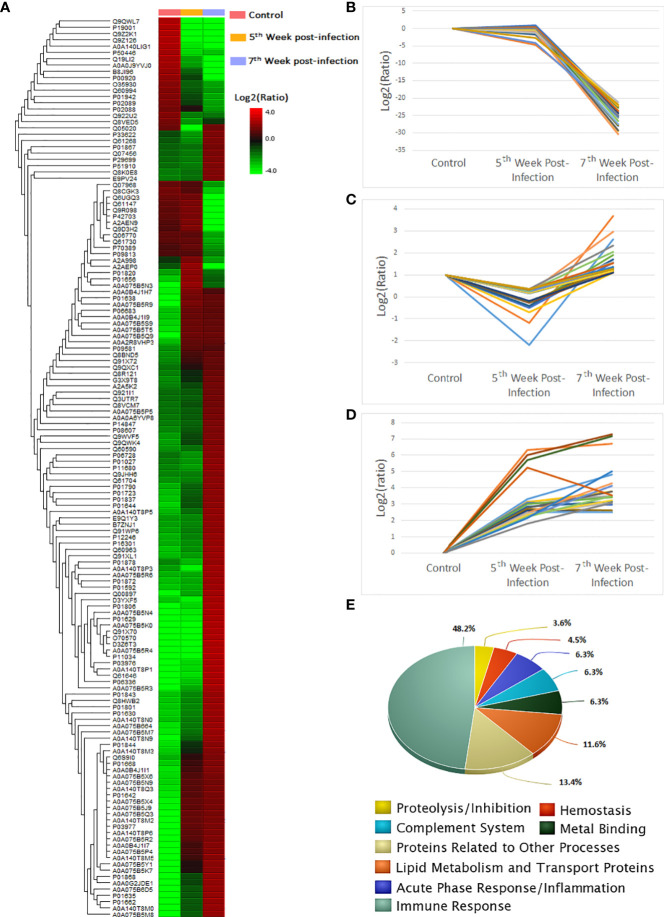
Heatmap of differentially abundant serum proteins in the context of *S. mansoni* infection in the murine model. **(A)** A total of 143 molecules with fold changes ≥ 2 were listed. Lipid transport and metabolism related proteins are marked in red. **(B-D)** Distinct expression patterns for differentially abundant serum constituents during *S. mansoni* infection. Molecules displaying downregulation in infected samples **(B)**; downregulation at week 5 post-infection followed by upregulation at week 7 **(C)**; upregulation in both time points **(D)**; list of theses molecule identities is seen on **Supplementary Table 3**. **(E)** Functional categorisation according to biological processes for differentially abundant serum constituents.

The quantitative label-free proteomic analysis revealed 143 shared constituents (31.5% of the total identified proteins) as differentially abundant comparing control and infected individuals. A consolidated list of the top three differentially abundant proteins categorised according to biological process is shown on **Table 1** (see **Supplementary Table 2** for a list of differentially abundant molecules). A heatmap plot indicated that over ⅔ of the differentially abundant proteins showed positive regulation at the 7^th^ week. The 5^th^ week period represented a transition with a few groups of proteins exhibiting either increased or decreased abundance, whilst for the majority of identities, levels did not differ significantly from control samples (**Figure 2A**). Only 22 proteins appeared significantly down regulated exclusively at 7 weeks (**Figure 2B**) and 36 displayed contrasting patterns of abundance at weeks 5 and 7 (**Figure 2C**). For the remaining differentially abundant proteins (85 molecules) during infection, increased levels relative to controls were observed at these two time points with higher abundance at 7^th^ week; possibly indicating that these molecules accumulated in the serum as the peak of the acute phase had been reached (**Figure 2D**). Protein lists associated with these distinct patterns of protein expression are provided in **Supplementary Table 3**.

**Table 1:** Consolidated list of the top three differentially abundant proteins categorised according to biological process.

Accession	Description	Coverage (%)	Molecular Mass (Da)	Log_2_ 5^th^ week/Control	Log_2_ 7^th^ week/Control	Significance
	**Lipid Metabolism and Transport Proteins (n=13)**					
Q60963	Platelet-activating factor acetylhydrolase	10	49258	1,11	3,36	67,75
P16301	Phosphatidylcholine-sterol acyltransferase	8	49747	0,60	2,76	64,11
Q9QWK4	CD5 antigen-like	42	38863	1,17	2,14	30,03
	**Acute Phase Response/Inflammation Proteins (n=7)**					
Q00897	Alpha-1-antitrypsin 1-4	45	45998	2,15	5,03	124,24
P12246	Serum amyloid P-component	45	26247	0,82	2,77	91,06
Q60590	Alpha-1-acid glycoprotein 1	28	23895	0,54	2,08	37,12
	**Immune Response Proteins (n=54)**					
P03976	Ig kappa chain V-II region 17S29.1	27	12390	3,64	7,77	116,64
A0A075B5P4	Ig gamma-1 chain C region secreted form	52	35752	4,27	4,90	119,12
A0A075B664	Immunoglobulin lambda variable 2	14	12165	2,22	4,11	14,21
	**Complement System Proteins (n=7)**					
D3YXF5	Complement component 7	6	93338	-1,19	3,67	96,63
P06683	Complement component C9	10	62002	2,63	2,51	32,24
P11680	Properdin	14	50327	-0,42	1,92	86,36
	**Metal Binding (n=7)**					
G3X9T8	Ceruloplasmin	49	121080	0,97	1,64	19,67
Q91X72	Hemopexin	60	51318	1,15	1,50	33,69
Q921I1	Serotransferrin	74	76724	0,20	1,18	28,33
	**Hemostasis (n=5)**					
E9PV24	Fibrinogen alpha chain	44	87429	0,19	1,60	51,35
Q8K0E8	Fibrinogen beta chain	77	54753	0,17	1,57	41,61
Q8VCM7	Fibrinogen gamma chain	73	49391	0,35	1,27	23,31
	**Proteolysis/Inhibition (n=15)**					
Q91WP6	Serine protease inhibitor A3N	43	46718	0,68	2,50	54,96
Q61704	Inter-alpha-trypsin inhibitor heavy chain H3	24	99358	0,27	2,06	42,84
Q9JHH6	Carboxypeptidase B2	5	48871	0,06	1,77	45,5
	**Proteins related to other processes (n=54)**					
Q91XL1	Leucine-rich HEV glycoprotein	24	37431	0,69	3,20	63,36
A0A2R8VHP3	Predicted pseudogene 5478	11	57920	2,49	2,50	26,63
Q9WVF5	Receptor protein-tyrosine kinase	7	72907	1,04	2,05	32,28

Significance set to ≥ 13 meaning p-value ≤ 0,05 (=-Log^10^(p-value)*10).

These proteins were further categorised according to the main biological processes they are related to (**Figure 2E**). Approximately half (48.21%, 54 molecules) of the differentially abundant molecules were associated with immune response. Among these, immunoglobulin kappa and lambda chains comprised the most prevalent signatures within this group. Immunoglobulin heavy chains belonging to isotypes IgM (mu chain), IgG (gamma chain), IgA (alpha chain) and 5 peptides matching the J chain, which joins two IgM or IgA monomers, confirmed the dominant immunoglobulin profile induced by the parasitic infection. For the majority of Ig entries, positive regulation was seen early at the 5^th^ week and fold changes relative to control were higher at the 7^th^ week. In the Acute Phase Response protein group, the classic C-Reactive Protein, Alpha-1B-glycoprotein and Serum amyloid P-component were the main representatives. In this group, positive regulation was observed at the 5^th^ week (except for Alpha-2-HS-glycoprotein), and increased expression was seen at the 7^th^ week.

Proteins related to lipid metabolism and transport were represented by 13 members. In this category, over 50% were apolipoproteins belonging to subtypes A-II, A-IV, B-100, C-II, C-III, C-IV, and D. Overall, apolipoproteins were up regulated at the peak of acute phase, with most exhibiting down regulation at the 5^th^ week. In this scenario, ApoB100 constitutes an exception, exhibiting a discrete increase (~30%) at the 5^th^ week and reaching approximately 500% increase at the 7^th^ week. In parallel, proteins involved in the control of fat metabolism and immune regulation, such as CD5 antigen-like and adiponectin displayed contrasting patterns of expression in response to the schistosome infection. For lipid transport proteins, whilst corticosteroid-binding globulin displayed down regulation as disease progressed from 5 to 7 weeks, phospholipid transfer protein exhibited an opposite pattern of abundance.

Among the differentially abundant proteins 7 members of the complement system contributed 6,25% to the total of identities (**Supplementary Table 2**). Equal contribution was provided by metal binding and acute phase response proteins. The former were mostly associated with iron binding and transport represented by ceruloplasmin, serotransferrin and hemopexin, all displaying increased abundance as infection progressed from 5 to 7 weeks. In agreement, decreased levels were seen for alpha and beta haemoglobin subunits. Protein identities related to hemostasis and proteolysis, or its inhibition, contributed <5% each in the total number of differentially abundant serum constituents. Concerning coagulation factors, fibrinogen alpha, beta and gamma chains were consistently up regulated from 5 to 7 weeks post infection. In contrast, coagulation factor XIII B chain and platelet glycoprotein Ib alpha chain exhibited down regulation at these two time points. Alpha-1-antitrypsin, also classified as an acute phase response protein, is a protease inhibitor for which expression levels were high at the 5^th^ week and continued to accumulate, as seen at the 7^th^ week. The same behaviour was shared by kininogen 2, inter-alpha trypsin inhibitor, protein Z-dependent protein inhibitor and the serine protease inhibitor A3N.

**Figure 3:**
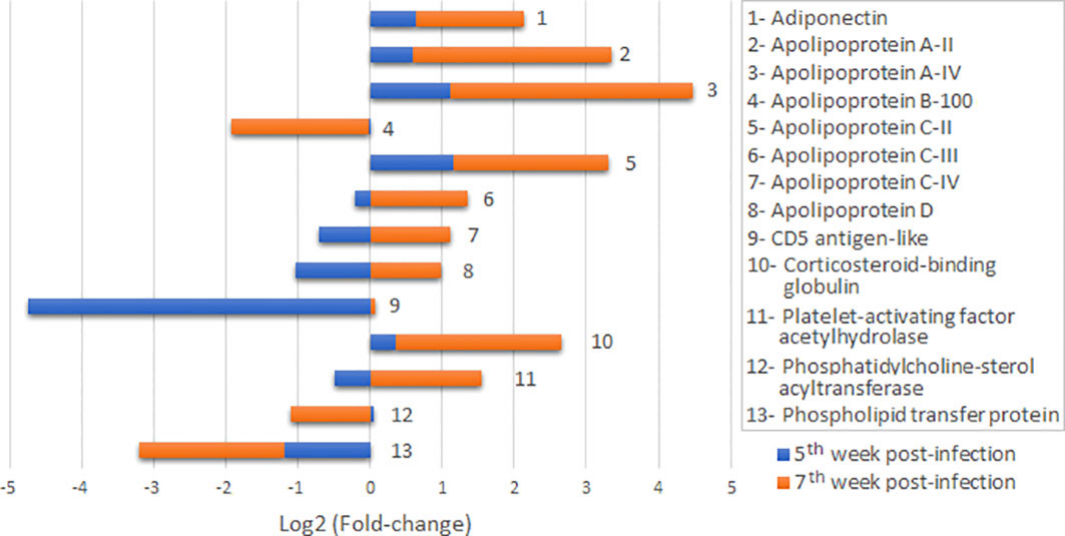
Quantitative label-free analysis plot for lipid metabolism and transport proteins at the 5^th^ and 7^th^ weeks post-infection. Protein identities are displayed in alphabetical order. Fold-changes are shown in vertical bars.

Of note, abundance profiles for lipid metabolism and transport proteins at the 5^th^ and 7^th^ weeks were quite divergent (**Figure 3**), typified by downregulation at 5^th^ week post-infection (except for platelet-activating factor acetylhydrolase), and up regulation at the 7^th^ week for the majority of identities in this group. Three exceptions for this behaviour were adiponectin, corticosteroid-binding globulin and apolipoprotein A-II, which displayed decreased abundance up to the peak of the acute phase in infected animals. In contrast, Apolipoprotein B-100, CD5-antigen like, platelet-activating factor acetylhydrolase, phosphatidylcholine sterol transferase and phospholipid transfer protein exhibited increased abundance throughout the investigated periods. Apolipoproteins C-III, C-IV and D were at reduced levels at week 5 in infected animals but their levels increased relative to non-infected individuals at week 7. In this group, Apolipoprotein C-II displayed the most pronounced fold decrease early at the 5^th^ week, exhibiting a discrete fold increase relative to control animals at the 7^th^ week.

**Figure 4:**
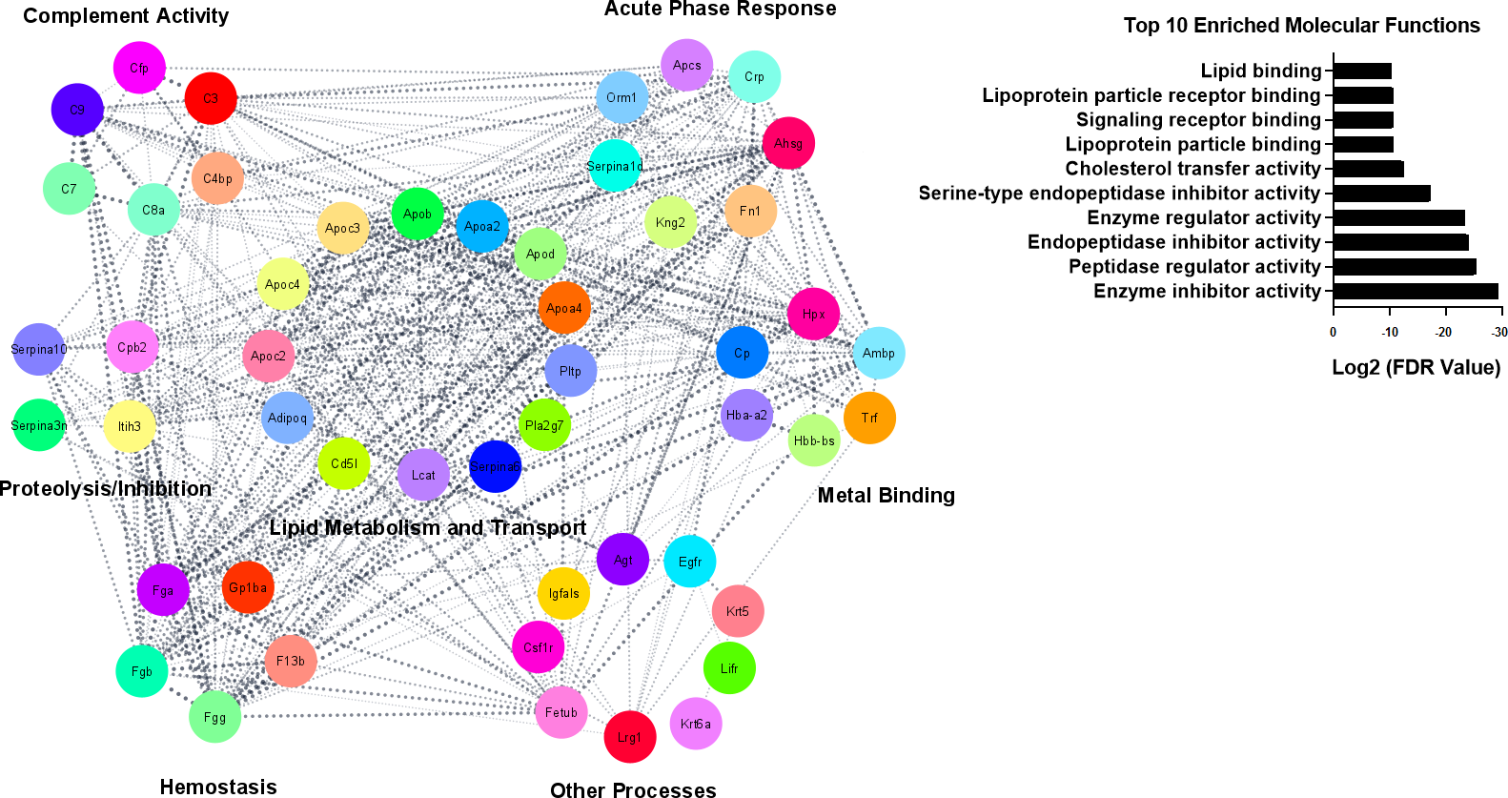
Protein interaction network displaying differentially abundant proteins, excluding immunoglobulins. Fifty out of 58 identities composed the interactome. Cytoscape Software v. 3.9.1, underpinned by STRING query tool for interrogation of the *Mus musculus* database, was used to visualise the generated network. Confidence cutoff score was set to > 0.4. Cytoscape also provided the Top 10 enriched molecular functions related to this network. The significant terms and pathways were selected with the threshold of adjusted Log2 FDR < -10. Abbreviations for protein identities are shown in **Supplementary Table 2**.

Given the molecular diversity in the serum proteome, 15 unrelated identities, accounting for approximately 14% of those showing altered levels, were grouped as belonging to various biological processes. Among them, known serum markers of cell proliferation (e.g., leucine rich HEV glycoprotein), angiotensin 1-10 and fetuin-B displayed up regulation at the peak of the acute phase. Next, the differentially abundant constituents (apart from immunoglobulins) were interrogated for previously reported protein-protein interactions using Cytoscape software (**Figure 4**). The retrieved interactome revealed a highly connected protein network not only linking constituents under the same category, but also displaying various possibilities for protein interactions among those from distinct biological processes. A list of top 10 enriched molecular functions associated with the interactome reinforced lipid metabolism and protease inhibition as significantly overrepresented processes. Proteins uniquely found in one of the two time points were listed in **Supplementary Table 4**. The majority of identities were also represented by immunoglobulin light and heavy chains plus other identities with no clear enrichment to merit their classification according to biological function. As they were not consistently identified in all of the samples, they were not dealt with any further in this study.

**Figure 5:**
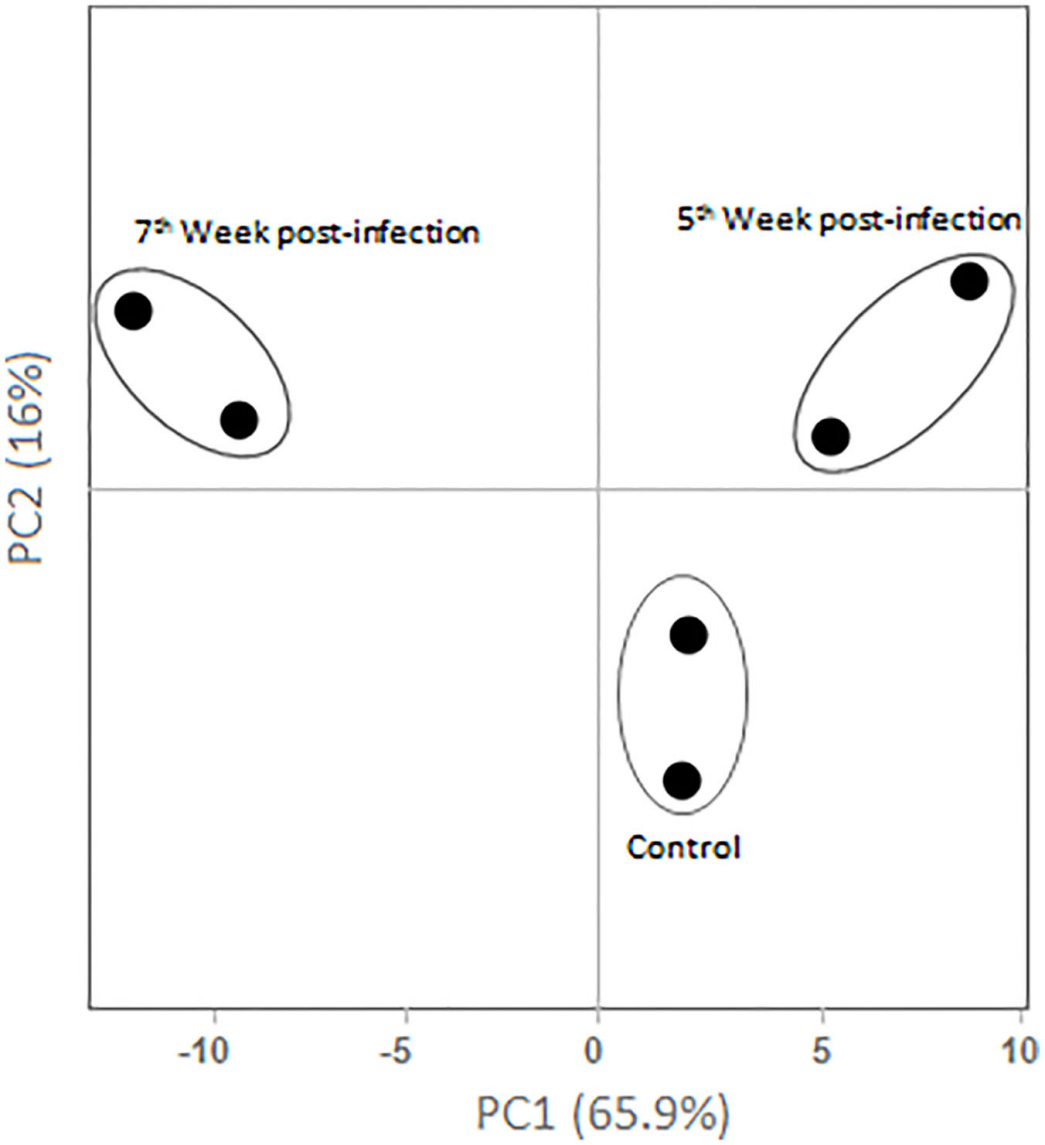
Principal Component Analysis. Unit variance scaling was applied, the standard variation with imputation was used to calculate principal components. Axes demonstrates principal component 1 and principal component 2 showing 65.9% and 16% of the total variance, respectively.

In light of the above findings concerning the distinct pattern of serum constituents, we wondered whether the protein profile associated with each condition (non-infected versus infected animals at the 5^th^ and 7^th^ weeks post infection) would allow segregation of individuals using principal component analysis. As observed in **Figure 5**, the PCA plot revealed a clear discrimination between groups. In agreement with the quantitative label-free analysis, serum profile from 5 week infected individuals was more closely related to that of control animals. The major fold differences observed for serum constituents at week 7 justifies its position in the PCA plot. This analysis demonstrated that a window of two weeks difference between evaluated samples in the acute phase of murine schistosomiasis promoted distinct profiles in the relative abundance of serum constituents.

### Serum lipid profile during acute schistosomiasis

**Figure 6:**
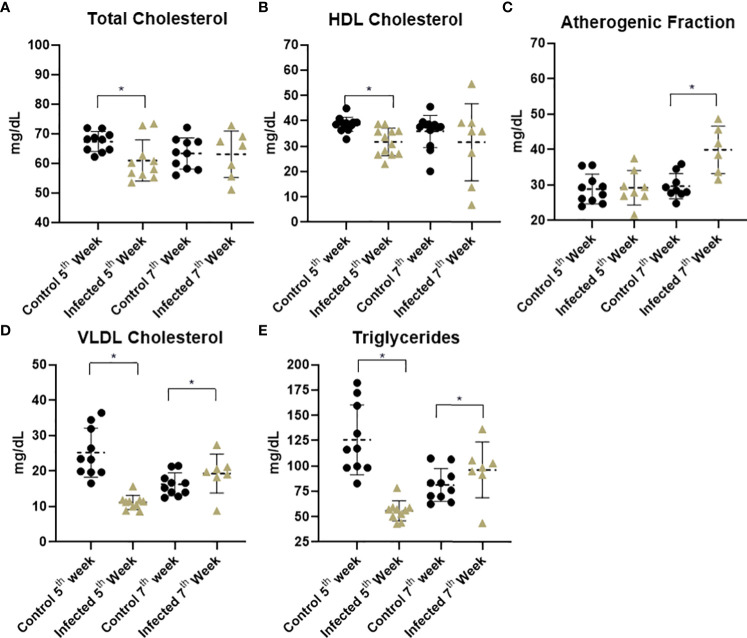
Serum lipid profile of experimental groups. Graphics represent the arithmetic means and standard deviations. Asterisks indicate significant differences between the evaluated conditions (p ≤ 0.05). **(A)** Total cholesterol content of control and infected mice. **(B)** HDL cholesterol demonstrated a decrease at 5^th^ week post infection. **(C)** Infected mice at 7^th^ week displayed a significant increase in the atherogenic fraction. **(D)** VLDL cholesterol levels declined at 5^th^ week post infection and increased at the late time point. **(E)** Triglyceride levels displayed a significant decrease at 5^th^ week post infection followed by an increase at the 7^th^ week.

In order to back up the obtained proteomic data and the observed altered levels for proteins related to lipid transport and metabolism, we performed a comparative evaluation of the lipid profile for control and infected individuals at the 5^th^ and 7^th^ weeks post-infection. Lipidogram analyses demonstrated significant alterations in the sera from infected individuals. Decreased levels for Total Cholesterol (**Figure 6A**) and HDL Cholesterol (**Figure 6B**) were observed at the 5^th^ week, with marked reduced levels for both VLDL Cholesterol and Triglycerides. At this time point, the non-HDL Cholesterol, here termed the atherogenic fraction (**Figure 6C**), remained unchanged. In the late investigated period (7^th^ week post infection), disturbances in circulating lipid molecules were mostly related to VLDL Cholesterol (**Figure 6D**) and Triglycerides (**Figure 6E**). At this time point, both Total and HDL Cholesterol levels did not differ significantly. Both evaluated periods exhibited significant changes in the circulating lipid-content of lipoproteins from infected animals, however, with distinct patterns associated with each time point. These results, expressed as mean ± standard deviation, are listed in **Supplementary Table 5**.

## Discussion

In this study, we used mass spectrometry-based label-free quantitative proteomic analyses to comparatively evaluate the serum proteome composition in two distinct phases of murine schistosomiasis. Firstly, at the 5^th^ week post-infection, representing a stage in which parasites have completed their sexual maturation with subsequent pairing and initiation of egg-laying by females. Secondly, at the 7^th^ week post-infection, meaning the peak of the acute phase, characterised by changes from a dominant T_h_1 to an egg-induced T_h_2 response and extensive granuloma formation, leading to impaired liver function (4). In this investigation, the spleen weight and spleen-to-body weight ratios for control and infected animals were in agreement with previous studies (9–11) allowing to ascertain that schistosomiasis has successfully been established in the murine model.

The obtained compositional analysis reflected the paramount challenge of protein discovery in serum samples. Since no fractionation methods were employed, the number of identified protein groups represented a reasonable achievement for a shotgun approach using non-depleted sera. Although a single molecule (albumin) comprised 73% of the cumulative abundance, the remaining identified proteins highlighted uncovered aspects of the *S. mansoni* parasitism concerning host-parasite interactions. This quantitative label-free proteomic analysis provided a repertoire of differentially abundant serum proteins over the course of *S. mansoni* experimental schistosomiasis up to the peak of its acute phase.

In our previous investigation using shotgun analysis of the soluble liver proteome, we have shown that it undergoes significant alteration in protein expression as schistosomiasis evolves (10). Those findings were also in agreement and complemented earlier findings using the 2D-DIGE approach applied to the murine model of this disease (8, 9). Taking into account that the liver is a secretory organ and its protein products constitute a major source of serum constituents, it was not surprising that relative levels in the serum for liver-derived molecules were significantly altered due to the infection. In the present study, we have identified classical acute-phase response proteins with altered levels early at week 5, but more pronounced alteration at week 7, as shown for C-reactive protein, alpha-1-acid glycoprotein and serum amyloid protein. Their altered levels in the serum of infected animals is possibly a non-specific response to the antigenic burden associated with the parasitism. In agreement, a recent plasma proteome study with patients on acute phase COVID-19 (16) and on infants that had been exposed to Zika Virus (17), demonstrated a similar set of molecules exhibiting altered levels in the plasma associated with both infectious diseases.

Almost one-third of the identified molecules showed altered levels in the serum during experimental schistosomiasis. Complement constituents, acute phase and immune response proteins were responsible for approximately 61% of those differentially abundant, with immunoglobulins representing the largest group in numbers. In this investigation peptides matching IgG have chains contributed with the majority of identities compared to IgA and IgM isotypes. The antigenic stimuli for the induced humoral response are associated with every aspect of the *S. mansoni* life cycle in the vertebrate host, from cercariae penetration (18) to its final establishment in the hepatic portal system (19, 20).

A novel aspect of this host-parasite interaction was revealed by the abundance profile of proteins related to lipid metabolism with implications for immune regulation. CD5 antigen-like is a key regulator of lipid synthesis and inflammatory response mainly secreted by macrophages (21). It circulates in the bloodstream in association with IgM (22). On adipose tissue, it is internalised by CD36 receptor and binds to fatty acid synthase promoting a decrease in its activity. As a result, lipolysis is stimulated leading to a decrease in lipid droplet content through mobilisation of triglycerides and consequent efflux of fatty acids and glycerol (23, 24). Hepatocytes also incorporate CD5 antigen-like through the same receptor (21, 25). In agreement, the soluble hepatic proteome evaluated during the acute phase of *S. mansoni* infection (10) also demonstrated downregulation of fatty acid synthase in addition to other lipid metabolism-related proteins. In our findings, CD5 antigen-like (Q9QWK4) exhibited increased abundance in both investigated time points. As a classically described immune effector, its expression is positively regulated under inflammatory circumstances (21), justifying its interpretation as a potential biomarker of liver damage (21, 26, 27). To our knowledge, we are the first to report on the differential abundance of CD5 antigen like in the sera of *S. mansoni* infected animals.

In contrast, adiponectin, a classical anti-inflammatory adipose tissue-secreted adipokine which also modulates macrophage function (28) exhibited down regulation in infected animals. Under normal circumstances, it promotes inhibition of inflammatory chemokines and regulates the production of anti-inflammatory cytokines (29). In addition, adiponectin decreases serum levels of triglycerides by enhancing lipoprotein lipase gene expression and activity, leading to decreased levels of circulating triglyceride-rich lipoproteins in the bloodstream (30–32). Moreover, adiponectin augments circulating HDL-C levels by stimulating ApoA-I hepatic synthesis and the ATP-Binding Cassette Transporter A1 pathway (33–35). The decreased abundance in circulating adiponectin observed in our study is in agreement with the inflammatory condition associated with the schistosome infection. The accompanying reduction in HDL cholesterol levels was particularly observed at week 5. At week 7, the sustained low levels of adiponectin are in line with the increased content of liver derived lipoproteins (mostly composing the atherogenic fraction), a finding reinforced by high levels of apolipoprotein B-100 at the two investigated time points.

Our quantitative data also points to differential lipoprotein remodelling associated with the schistosome infection. Not only lipoproteins were found mainly upregulated, the enzymes phospholipid transfer protein and phosphatidylcholine-sterol acyltransferase, known to participate in the transfer and chemical modification of their lipid contents, increased in abundance as judged by their relative levels at weeks 5 and 7 in infected animals. Of note, various differentially abundant constituents, such as those involved in acute phase response, complement cascade and proteinase inhibitors, have been reported to be ligand-binding partners of lipoproteins, in particular HDL (36, 37). In fact, serum amyloid protein, here found upregulated, is currently considered an exchangeable lipoprotein. It was demonstrated to be associated with VLDL, LDL and HDL leading to enhanced binding of these particles to surface proteoglycans (38). Protocols designed to enrich for lipoproteins in the context of schistosomiasis should provide a better view of their potential ligands and clues to their distinct roles in immune response or sustaining parasitism. Concerning the latter, a recent report has highlighted the lipidomic profile in the *S. mansoni* -infected hamster liver using a combination of mass spectrometric approaches (39). Over 300 lipid markers of infection were appointed in the granulomatous liver, mostly representing phosphatidylcholines, phosphatidylethanolamines, and triglycerides. Our findings provide molecular clues into how lipid metabolism is modulated to attend both host and parasite demands for lipid and lipid-derived molecules. In agreement, the protein-protein interaction network, revealed the potential for complex interactions among molecules related to Lipid Metabolism and Transport group with those belonging to other categories such as acute phase response, complement activity, proteolysis inhibition and hemostasis. Given the predominance of proteins with increased abundance composing this interactome, the various binding possibilities attests for intricate mechanisms of immune response and host metabolism operating in the bloodstream during *S. mansoni* infection.

**Figure 7:**
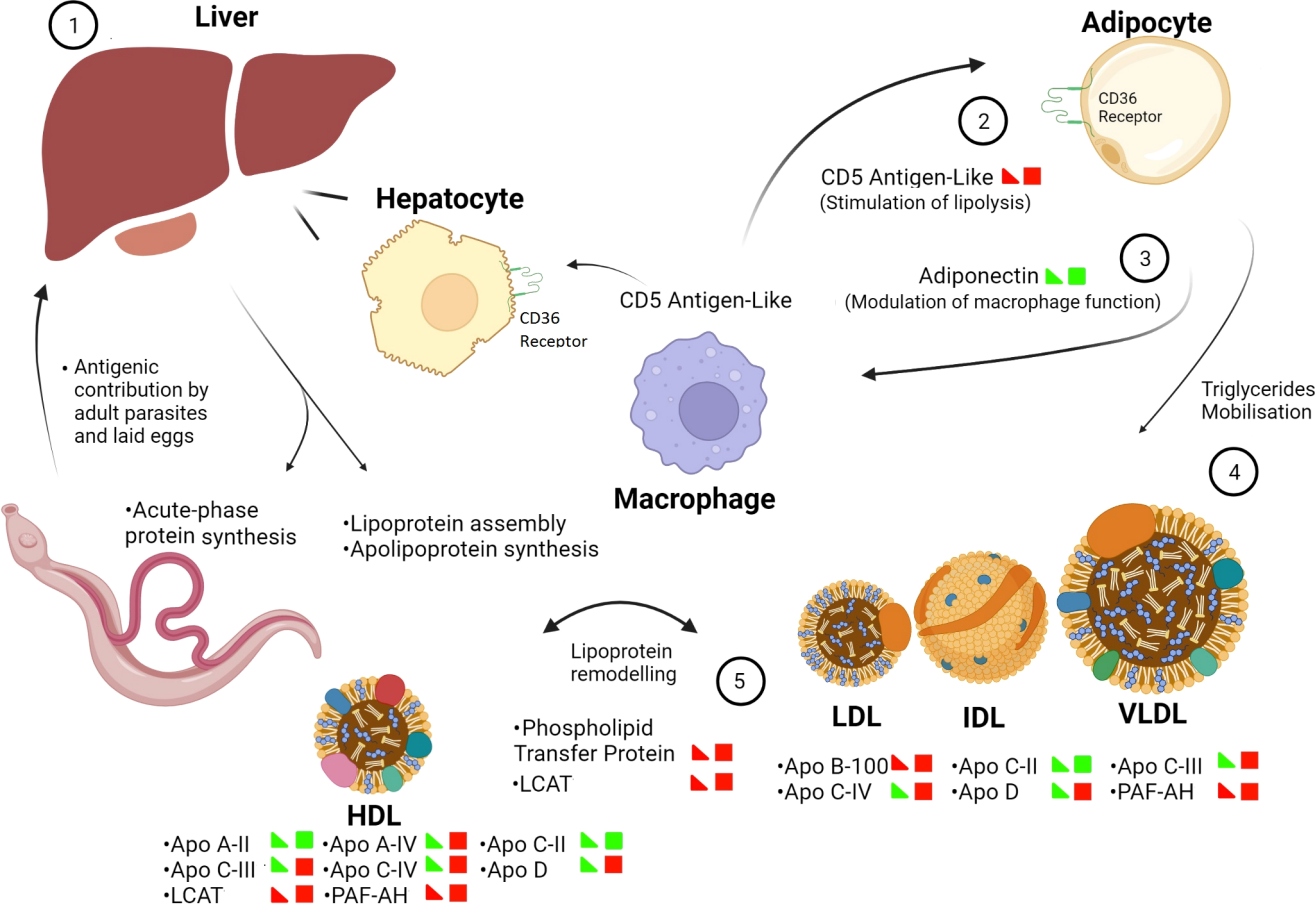
Proposed model for lipid metabolism regulation during schistosomiasis and the influence of macrophage and adipose tissue-secreted molecules. Up or down regulation of proteins are represented by half and full squares, meaning the 5^th^ and 7^th^ week post-infection periods, respectively. Colored squares indicate downregulation (green) or upregulation (red). 1) The liver responsible for lipoprotein assembly, apolipoprotein synthesis and overall metabolism receives the antigenic burden associated with adult parasites and laid eggs leading to pronounced changes in protein expression. Macrophages are a hallmark of inflammation during schistosomiasis 2) CD5 antigen-like, mostly secreted by macrophages, can be internalised on hepatocytes and adipocytes through CD36 receptors. 3) Reduced levels of adiponectin secreted by adipocytes contribute to modulate macrophage function and maintenance of the inflammatory status. Adiponectin also influences the serum levels of HDL-C and triglycerides. 4) CD5 antigen-like stimulates lipolysis with consequent triglyceride mobilisation. 5) Altered levels of apolipoproteins and upregulated levels of lipoprotein modifying enzymes (phospholipid transfer protein and LCAT) suggests pronounced lipoprotein remodelling during *S. mansoni* infection. Created with BioRender.com.

In this study, quantitative label-free proteomic analysis revealed lipid metabolism related proteins as key modulators of host-parasite interactions during the acute phase of schistosomiasis (**Figure 7**). The antigenic burden associated with the schistosome infection, in particular during the onset of egg laying, induces an inflammatory condition possibly linked to intense mobilisation of lipids as reflected in the altered lipoprotein profile and proteome of the infected sera. Our study has demonstrated macrophage and adipose tissue-secreted molecules, apolipoproteins/lipoprotein-bound proteins and enzymes differentially abundant, possibly contributing to lipid metabolism modulation. The complex interactions among the differentially abundant serum constituents and target tissues points to intense lipid metabolism regulation during schistosomiasis. It remains to be determined whether the seen alterations in the mouse serum can be translated to human disease to explore novel markers of active infection and inform on the inflammatory status posed by the *S. mansoni* parasitism.

## Data availability statement

The mass spectrometry proteomics data have been deposited to the ProteomeXchange Consortium via the PRIDE (40) partner repository with the dataset identifier PXD031974.

## Ethics statement

The experimental procedures involving mice were reviewed and approved by the local Ethics Committee on Animal Experimentation ‘Comissão de Ética no Uso de Animais’ (CEUA), Universidade Federal de Ouro Preto (UFOP), and received the protocol numbers 2017/35 and 2017/34, respectively.

## Author contributions

GG-S and WC-B designed, analysed, interpreted data and wrote the manuscript. GG-S and LSV conducted experiments. MC-C and AS conducted instrumental analysis and provided technical expertise. DC contributed to the interpretation of data. All authors reviewed the manuscript. All authors contributed to the article and approved the submitted version.

## Funding

This study was financially supported by Fundação de Amparo à Pesquisa do Estado de Minas Gerais (FAPEMIG), grant number: APQ-02745-18, FAPEMIG - Rede Mineira de Imunobiológicos, grant number: 00140-16, Conselho Nacional de Desenvolvimento Científico e Tecnológico (CNPq) grant number: 438798/2018-0 and Universidade Federal de Ouro Preto (UFOP), grant number: 23109.000928/2020-33. GG-S is a recipient of a scholarship from Coordenação de Aperfeiçoamento de Pessoal de Nível Superior - Brasil (CAPES), Finance Code 001. WC-B is a fellow researcher from CNPq (311286/2019-4). The authors also acknowledge the support provided by PROPPI/UFOP and CAPES for fundings provided through the internal call number 18/2022.

## Acknowledgments

The authors acknowledge Lobato Paraense Mollusk facility at René Rachou (Fiocruz Minas) for providing the *S. mansoni* cercariae. The BioClin-Quibasa for providing the reagent kits for serum lipids analysis and Laboratório Piloto de Análises Clínicas (Lapac-UFOP). The authors also acknowledge the Laboratório Multiusuário de Proteômica e Biomoléculas (LMU-ProtBio), from Núcleo de Pesquisas em Ciências Biológicas, Universidade Federal de Ouro Preto, MG, Brazil for providing the required equipments and technical expertise.

## Conflict of interest

The authors declare that the research was conducted in the absence of any commercial or financial relationships that could be construed as a potential conflict of interest.

## Publisher’s note

All claims expressed in this article are solely those of the authors and do not necessarily represent those of their affiliated organizations, or those of the publisher, the editors and the reviewers. Any product that may be evaluated in this article, or claim that may be made by its manufacturer, is not guaranteed or endorsed by the publisher.
