# Supplementary material for: Profiling the serum proteome during Schistosoma mansoni infection in the BALB/c mice: A focus on the altered lipid metabolism as a key modulator of host-parasite interactions
Source: Front Immunol. 2022 Aug 31;13:955049. doi: 10.3389/fimmu.2022.955049 (PMC9471378; doi:10.3389/fimmu.2022.955049)
Supplement: Supplementary file 1 [file DataSheet_1.docx]

Supplementary Material

# Supplementary Data

# Supplementary Figures and Tables


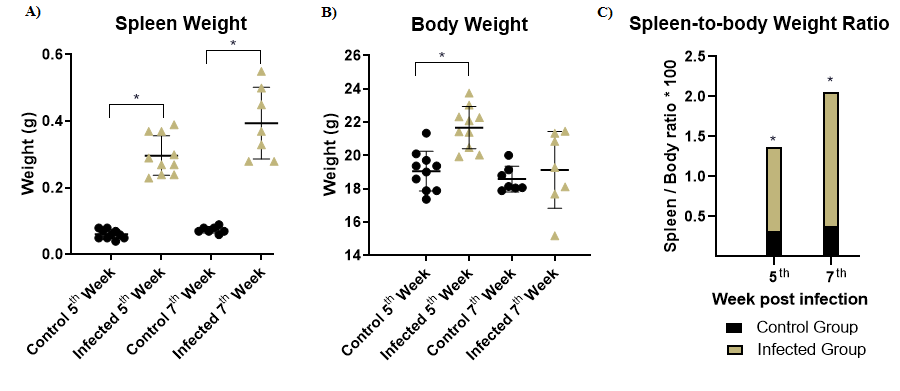


**Supplementary Figure 1.** Establishment of *S. mansoni* infection in the BALB/c mice. Graphics indicate the arithmetic means and standard deviation. Asterisks indicate significant differences between the evaluated conditions (p≤0.001). **A**) Mice spleen weight throughout the investigated weeks post infection. **B**) Body weight of infected groups at the 5^th^ and the 7^th^ weeks post infection. **C**) Spleen-to-body weight ratios for infected and control animals.

**Supplementary Tables**

Supplementary Table 1. Compositional analysis of the serum proteome during *S. mansoni* infection.

| **Accession** | **Description** | **Molecular mass (Da)** | **Coverage (%)** | **Identified Peptides** | **Unique peptides** |
| --- | --- | --- | --- | --- | --- |
| O70456 | 14-3-3 protein sigma | 27706 | 11 | 3 | 1 |
| P63101 | 14-3-3 protein zeta/delta | 27771 | 13 | 3 | 1 |
| Q91UZ1 | 1-phosphatidylinositol 4 5-bisphosphate phosphodiesterase | 134527 | 1 | 1 | 1 |
| O35176 | ABPA2 | 10288 | 48 | 4 | 4 |
| P63260 | Actin cytoplasmic 2 | 41793 | 55 | 17 | 16 |
| G3UYQ4 | Adenylate kinase 9 | 219532 | 0 | 1 | 1 |
| Q60994 | Adiponectin | 26809 | 19 | 4 | 4 |
| O89020 | Afamin | 69379 | 53 | 34 | 34 |
| A0A140LJJ5 | A-kinase anchor protein 13 | 306005 | 0 | 1 | 1 |
| P00329 | Alcohol dehydrogenase 1 | 39771 | 2 | 1 | 1 |
| Q60590 | Alpha-1-acid glycoprotein 1 | 23895 | 30 | 9 | 7 |
| P07361 | Alpha-1-acid glycoprotein 2 | 23843 | 18 | 5 | 3 |
| P22599 | Alpha-1-antitrypsin 1-2 | 45975 | 60 | 21 | 7 |
| Q00896 | Alpha-1-antitrypsin 1-3 | 45823 | 56 | 24 | 1 |
| Q00897 | Alpha-1-antitrypsin 1-4 | 45998 | 51 | 20 | 3 |
| Q19LI2 | Alpha-1B-glycoprotein | 56554 | 20 | 12 | 12 |
| Q61247 | Alpha-2-antiplasmin | 54972 | 48 | 21 | 21 |
| P29699 | Alpha-2-HS-glycoprotein | 37326 | 65 | 17 | 17 |
| Q6GQT1 | Alpha-2-macroglobulin-P | 164352 | 5 | 7 | 3 |
| P00687 | Alpha-amylase 1 | 57644 | 9 | 3 | 3 |
| P11859 | Angiotensinogen | 51990 | 27 | 8 | 8 |
| G5E893 | Ankyrin repeat domain 12 | 232910 | 0 | 1 | 1 |
| P07356 | Annexin A2 | 38676 | 26 | 7 | 7 |
| P32261 | Antithrombin-III | 52004 | 56 | 30 | 30 |
| Q00623 | Apolipoprotein A-I | 30616 | 77 | 29 | 29 |
| P09813 | Apolipoprotein A-II | 11309 | 15 | 2 | 2 |
| P06728 | Apolipoprotein A-IV | 45029 | 85 | 39 | 39 |
| E9Q414 | Apolipoprotein B-100 | 509437 | 22 | 78 | 77 |
| P34928 | Apolipoprotein C-I | 9696 | 36 | 5 | 4 |
| Q05020 | Apolipoprotein C-II | 10741 | 38 | 4 | 4 |
| P33622 | Apolipoprotein C-III | 10982 | 55 | 5 | 5 |
| Q61268 | Apolipoprotein C-IV | 14288 | 22 | 3 | 3 |
| P51910 | Apolipoprotein D | 21530 | 31 | 6 | 6 |
| P08226 | Apolipoprotein E | 35867 | 53 | 21 | 20 |
| Q9Z1R3 | Apolipoprotein M | 21273 | 41 | 9 | 8 |
| G3X9D6 | Apolipoprotein N | 27965 | 7 | 3 | 3 |
| D3YX85 | Arf-GAP with SH3 domain ANK repeat and PH domain-containing protein 2 | 106405 | 1 | 1 | 1 |
| P05202 | Aspartate aminotransferase mitochondrial | 47411 | 2 | 1 | 1 |
| B7ZCC4 | Ataxin 7-like 2 | 69999 | 3 | 1 | 1 |
| O70133 | ATP-dependent RNA helicase A | 149474 | 1 | 1 | 1 |
| Q99PU8 | ATP-dependent RNA helicase DHX30 | 136668 | 1 | 1 | 1 |
| Q8CGN4 | BCL-6 corepressor | 192630 | 1 | 1 | 1 |
| Q01339 | Beta-2-glycoprotein 1 | 38619 | 63 | 22 | 22 |
| P01887 | Beta-2-microglobulin | 13779 | 21 | 3 | 3 |
| P97929 | Breast cancer type 2 susceptibility protein homolog | 370666 | 0 | 1 | 1 |
| P08607 | C4b-binding protein | 51524 | 27 | 10 | 10 |
| Q3UKW2 | Calmodulin-1 | 21560 | 9 | 1 | 1 |
| Q8C1B1 | Calmodulin-regulated spectrin-associated protein 2 | 164332 | 0 | 1 | 1 |
| P00920 | Carbonic anhydrase 2 | 29033 | 11 | 3 | 3 |
| P23953 | Carboxylesterase 1C | 61056 | 32 | 15 | 8 |
| D3Z5G7 | Carboxylic ester hydrolase | 62197 | 25 | 13 | 7 |
| Q9JHH6 | Carboxypeptidase B2 | 48871 | 13 | 5 | 5 |
| Q9JJN5 | Carboxypeptidase N catalytic chain | 51845 | 36 | 13 | 13 |
| Q9DBB9 | Carboxypeptidase N subunit 2 | 60479 | 37 | 16 | 16 |
| Q9WVJ3 | Carboxypeptidase Q | 51813 | 15 | 4 | 4 |
| P10605 | Cathepsin B | 37280 | 20 | 5 | 5 |
| D3Z6T3 | Cathepsin E | 39247 | 5 | 2 | 2 |
| O70370 | Cathepsin S | 38475 | 9 | 2 | 2 |
| Q9QWK4 | CD5 antigen-like | 38863 | 55 | 20 | 20 |
| Q9CZX2 | Centrosomal protein of 89 kDa | 90320 | 2 | 1 | 1 |
| G3X9T8 | Ceruloplasmin | 121080 | 53 | 56 | 1 |
| Q61147 | Ceruloplasmin | 121151 | 53 | 56 | 1 |
| Q61410 | cGMP-dependent protein kinase 2 | 87085 | 2 | 1 | 1 |
| Q9EQI5 | Chemokine subfamily B Cys-X-Cys | 12252 | 10 | 1 | 1 |
| E9Q2I1 | Chloride channel protein | 93665 | 1 | 1 | 1 |
| Q6AW69 | Cingulin-like protein 1 | 148230 | 1 | 1 | 1 |
| Q80YR7 | Claspin | 146715 | 1 | 1 | 1 |
| Q06890 | Clusterin | 51656 | 40 | 21 | 21 |
| P16294 | Coagulation factor IX | 52978 | 4 | 2 | 2 |
| O88783 | Coagulation factor V | 247228 | 2 | 3 | 3 |
| O88947 | Coagulation factor X | 54018 | 32 | 14 | 14 |
| Q80YC5 | Coagulation factor XII | 65701 | 18 | 9 | 9 |
| Q8BH61 | Coagulation factor XIII A chain | 83207 | 12 | 9 | 8 |
| Q07968 | Coagulation factor XIII B chain | 76195 | 21 | 12 | 12 |
| E9PX94 | Coiled-coil and C2 domain-containing protein 1A | 98643 | 1 | 1 | 1 |
| Q6PHN1 | Coiled-coil domain-containing protein 57 | 116194 | 1 | 1 | 1 |
| P14106 | Complement C1q subcomponent subunit B | 26717 | 18 | 4 | 4 |
| Q8CG16 | Complement C1r-A subcomponent | 80073 | 5 | 3 | 3 |
| P01027 | Complement C3 | 186483 | 78 | 125 | 124 |
| P01029 | Complement C4-B | 192914 | 32 | 48 | 48 |
| P06684 | Complement C5 | 188877 | 43 | 59 | 59 |
| E9Q6C2 | Complement component 1s subcomponent 1 | 77500 | 5 | 3 | 3 |
| Q91X70 | Complement component 6 | 86631 | 10 | 5 | 5 |
| D3YXF5 | Complement component 7 | 93338 | 13 | 8 | 8 |
| A2A998 | Complement component C8 alpha chain | 61008 | 27 | 11 | 11 |
| Q8BH35 | Complement component C8 beta chain | 66229 | 17 | 8 | 8 |
| Q9DAC2 | Complement component C8 gamma chain | 18946 | 51 | 6 | 6 |
| P06683 | Complement component C9 | 62002 | 26 | 14 | 14 |
| P03953 | Complement factor D | 28057 | 34 | 7 | 6 |
| P06909 | Complement factor H | 139137 | 64 | 64 | 50 |
| Q61129 | Complement factor I | 67261 | 66 | 36 | 36 |
| Q62266 | Cornifin-A | 15765 | 6 | 1 | 1 |
| Q62267 | Cornifin-B | 16636 | 5 | 1 | 1 |
| Q06770 | Corticosteroid-binding globulin | 44769 | 31 | 14 | 14 |
| P14847 | C-reactive protein | 25360 | 10 | 2 | 2 |
| Q9DB77 | Cytochrome b-c1 complex subunit 2 mitochondrial | 48235 | 2 | 1 | 1 |
| A2AF67 | Dedicator of cytokinesis protein 11 | 218242 | 1 | 2 | 1 |
| A0A5F8MPL9 | Dedicator of cytokinesis protein 9 | 235340 | 1 | 2 | 1 |
| E9Q557 | Desmoplakin | 332913 | 0 | 1 | 1 |
| E9Q9I2 | Disks large homolog 5 | 212095 | 0 | 1 | 1 |
| Q3U1J4 | DNA damage-binding protein 1 | 126853 | 1 | 1 | 1 |
| A2AG30 | DnaJ homolog subfamily B member 5 | 43018 | 2 | 1 | 1 |
| A0A140LIN9 | Dynein heavy chain 3 axonemal | 466723 | 0 | 1 | 1 |
| A0A171EBL2 | E3 ubiquitin-protein ligase RNF213 | 584635 | 1 | 2 | 2 |
| Q8BPB5 | EGF-containing fibulin-like extracellular matrix protein 1 | 54953 | 8 | 3 | 3 |
| P62631 | Elongation factor 1-alpha 2 | 50454 | 2 | 1 | 1 |
| Q01279 | Epidermal growth factor receptor | 134853 | 11 | 10 | 10 |
| Q9Z0N2 | Eukaryotic translation initiation factor 2 subunit 3 Y-linked | 51131 | 4 | 2 | 1 |
| E9Q770 | Eukaryotic translation initiation factor 4 gamma 1 (Fragment) | 103898 | 2 | 2 | 1 |
| A0A2I3BRQ1 | Expressed sequence AI182371 | 40054 | 16 | 5 | 5 |
| Q61508 | Extracellular matrix protein 1 | 62832 | 20 | 10 | 10 |
| O09164 | Extracellular superoxide dismutase [Cu-Zn] | 27392 | 11 | 2 | 2 |
| Q05816 | Fatty acid-binding protein 5 | 15137 | 7 | 1 | 1 |
| Q9QXC1 | Fetuin-B | 42713 | 49 | 15 | 15 |
| E9PV24 | Fibrinogen alpha chain | 87429 | 55 | 43 | 42 |
| Q8K0E8 | Fibrinogen beta chain | 54753 | 78 | 38 | 38 |
| Q8VCM7 | Fibrinogen gamma chain | 49391 | 74 | 33 | 33 |
| F8WH29 | Fibrocystin-L | 464508 | 0 | 1 | 1 |
| A0A087WSN6 | Fibronectin | 253009 | 53 | 81 | 68 |
| A2ARZ3 | Fibrous sheath-interacting protein 2 | 784873 | 0 | 1 | 1 |
| Q08879 | Fibulin-1 | 78033 | 11 | 5 | 5 |
| O70165 | Ficolin-1 | 36298 | 12 | 4 | 4 |
| F8WGX0 | FYVE RhoGEF and PH domain-containing protein 4 (Fragment) | 85245 | 1 | 1 | 1 |
| P13020 | Gelsolin | 85942 | 48 | 35 | 35 |
| A0A2I3BR94 | Glia maturation factor beta (Fragment) | 15916 | 4 | 1 | 1 |
| P26443 | Glutamate dehydrogenase 1 mitochondrial | 61337 | 2 | 1 | 1 |
| P46412 | Glutathione peroxidase 3 | 25424 | 43 | 10 | 9 |
| S4R257 | Glyceraldehyde-3-phosphate dehydrogenase (Fragment) | 29939 | 13 | 3 | 3 |
| P01898 | H-2 class I histocompatibility antigen Q10 alpha chain | 37251 | 46 | 14 | 8 |
| Q05A75 | H-2 class I histocompatibility antigen TLA(B) alpha chain | 43544 | 8 | 2 | 1 |
| Q61646 | Haptoglobin | 38752 | 67 | 23 | 23 |
| E9PYX1 | Harmonin | 100256 | 1 | 1 | 1 |
| G3X9B1 | HEAT repeat-containing 1 | 242069 | 0 | 1 | 1 |
| Q504P4 | Heat shock cognate 71 kDa protein | 68779 | 2 | 1 | 1 |
| P01942 | Hemoglobin subunit alpha | 15085 | 61 | 8 | 8 |
| P02088 | Hemoglobin subunit beta-1 | 15840 | 84 | 11 | 5 |
| P02089 | Hemoglobin subunit beta-2 | 15878 | 84 | 12 | 6 |
| Q91X72 | Hemopexin | 51318 | 63 | 33 | 33 |
| P49182 | Heparin cofactor 2 | 54497 | 36 | 16 | 16 |
| Q9R098 | Hepatocyte growth factor activator | 70568 | 17 | 7 | 7 |
| Q9ESB3 | Histidine-rich glycoprotein | 59163 | 30 | 17 | 17 |
| Q8HWB2 | Histocompatibility 2 Q region locus 4 | 39618 | 29 | 9 | 2 |
| P62806 | Histone H4 | 11367 | 27 | 3 | 3 |
| P55200 | Histone-lysine N-methyltransferase 2A | 429653 | 0 | 2 | 2 |
| Q8BRH4 | Histone-lysine N-methyltransferase 2C | 540193 | 0 | 1 | 1 |
| H3BK67 | Homeobox-containing protein 1 | 45920 | 2 | 1 | 1 |
| Q3V1J8 | Hyaluronan-binding protein 2 | 57326 | 4 | 2 | 2 |
| Q80W93 | Hydrocephalus-inducing protein | 581532 | 1 | 3 | 3 |
| P01878 | Ig alpha chain C region | 36876 | 45 | 10 | 9 |
| P06336 | Ig epsilon chain C region | 47321 | 41 | 13 | 13 |
| A0A075B5P4 | Ig gamma-1 chain C region secreted form (Fragment) | 35752 | 55 | 14 | 1 |
| P01868 | Ig gamma-1 chain C region secreted form | 35705 | 60 | 16 | 3 |
| P01863 | Ig gamma-2A chain C region A allele | 36389 | 42 | 12 | 7 |
| P01867 | Ig gamma-2B chain C region | 44259 | 37 | 13 | 1 |
| P18531 | Ig heavy chain V region 3-6 | 13095 | 19 | 2 | 2 |
| P01806 | Ig heavy chain V region 441 | 12911 | 72 | 9 | 1 |
| P18528 | Ig heavy chain V region 6.96 | 11007 | 49 | 5 | 1 |
| P18527 | Ig heavy chain V region 914 | 10661 | 46 | 5 | 2 |
| P01790 | Ig heavy chain V region M511 | 13652 | 57 | 6 | 5 |
| P01756 | Ig heavy chain V region MOPC 104E | 12983 | 53 | 4 | 2 |
| P01812 | Ig heavy chain V region MOPC 173 | 13051 | 58 | 8 | 1 |
| P01741 | Ig heavy chain V region | 12555 | 11 | 1 | 1 |
| P18524 | Ig heavy chain V region RF | 12866 | 33 | 5 | 1 |
| P01811 | Ig heavy chain V region UPC10 | 13001 | 37 | 5 | 2 |
| P01801 | Ig heavy chain V-III region J606 | 12810 | 49 | 6 | 1 |
| P01633 | Ig kappa chain V19-17 | 16434 | 38 | 6 | 1 |
| P01632 | Ig kappa chain V-I region S107A | 12717 | 18 | 3 | 1 |
| P03976 | Ig kappa chain V-II region 17S29.1 | 12390 | 50 | 3 | 2 |
| P01631 | Ig kappa chain V-II region 26-10 | 12273 | 48 | 5 | 1 |
| P01629 | Ig kappa chain V-II region 2S1.3 | 12221 | 35 | 2 | 2 |
| P01630 | Ig kappa chain V-II region 7S34.1 | 12496 | 26 | 3 | 2 |
| P01628 | Ig kappa chain V-II region MOPC 511 | 12496 | 19 | 2 | 1 |
| P03977 | Ig kappa chain V-III region 50S10.1 | 12042 | 46 | 4 | 1 |
| P01662 | Ig kappa chain V-III region ABPC 22/PC 9245 | 12041 | 65 | 5 | 1 |
| P01656 | Ig kappa chain V-III region MOPC 70 | 11904 | 68 | 5 | 1 |
| P01654 | Ig kappa chain V-III region PC 2880/PC 1229 | 11980 | 68 | 5 | 1 |
| P01660 | Ig kappa chain V-III region PC 3741/TEPC 111 | 12099 | 65 | 6 | 1 |
| P01670 | Ig kappa chain V-III region PC 6684 | 12039 | 70 | 5 | 1 |
| P01665 | Ig kappa chain V-III region PC 7043 | 12002 | 59 | 4 | 3 |
| P01644 | Ig kappa chain V-V region HP R16.7 | 11910 | 68 | 7 | 3 |
| P01652 | Ig kappa chain V-V region J606 | 11810 | 19 | 2 | 1 |
| P01635 | Ig kappa chain V-V region K2 (Fragment) | 12581 | 41 | 4 | 2 |
| P01638 | Ig kappa chain V-V region L6 (Fragment) | 12986 | 45 | 5 | 1 |
| P01642 | Ig kappa chain V-V region L7 (Fragment) | 12615 | 29 | 3 | 3 |
| P01636 | Ig kappa chain V-V region MOPC 149 | 12030 | 39 | 4 | 1 |
| P01639 | Ig kappa chain V-V region MOPC 41 | 14311 | 36 | 5 | 3 |
| P04940 | Ig kappa chain V-VI region NQ2-17.4.1 | 11561 | 38 | 4 | 1 |
| P01678 | Ig kappa chain V-VI region SAPC 10 | 11554 | 42 | 4 | 2 |
| P01843 | Ig lambda-1 chain C region | 11575 | 81 | 5 | 5 |
| P01723 | Ig lambda-1 chain V region | 12222 | 36 | 2 | 1 |
| P01725 | Ig lambda-1 chain V region S178 | 11654 | 21 | 1 | 1 |
| P01844 | Ig lambda-2 chain C region | 11255 | 86 | 6 | 6 |
| A0A075B5P5 | Immunoglobulin heavy constant gamma 3 (Fragment) | 36317 | 63 | 15 | 12 |
| P01872 | Immunoglobulin heavy constant mu | 49972 | 60 | 26 | 26 |
| A0A0B4J1J6 | Immunoglobulin heavy variable 10-1 (Fragment) | 13550 | 32 | 5 | 4 |
| A0A075B5R6 | Immunoglobulin heavy variable 11-1 (Fragment) | 13208 | 45 | 4 | 4 |
| A0A0A6YXA5 | Immunoglobulin heavy variable 1-15 (Fragment) | 12953 | 42 | 3 | 1 |
| A0A075B5R4 | Immunoglobulin heavy variable 14-1 (Fragment) | 12992 | 26 | 3 | 1 |
| A0A075B5V8 | Immunoglobulin heavy variable 1-47 | 11083 | 13 | 1 | 1 |
| A0A075B680 | Immunoglobulin heavy variable 1-62-2 | 11225 | 33 | 4 | 2 |
| A0A0B4J1M0 | Immunoglobulin heavy variable 1-77 | 10662 | 42 | 3 | 1 |
| A0A075B5Y4 | Immunoglobulin heavy variable 1-81 (Fragment) | 12989 | 26 | 3 | 1 |
| A0A0B4J1J7 | Immunoglobulin heavy variable 1-82 | 10650 | 20 | 2 | 1 |
| A0A075B5Q3 | Immunoglobulin heavy variable 2-5 | 12557 | 39 | 4 | 4 |
| A0A075B6A7 | Immunoglobulin heavy variable 2-6 (Fragment) | 12596 | 19 | 2 | 1 |
| A0A075B697 | Immunoglobulin heavy variable 2-9-1 (Fragment) | 12481 | 19 | 3 | 2 |
| A0A075B5Q4 | Immunoglobulin heavy variable 5-12 (Fragment) | 13075 | 37 | 5 | 1 |
| A0A075B5Q9 | Immunoglobulin heavy variable 5-15 | 12943 | 37 | 4 | 1 |
| A0A075B5R1 | Immunoglobulin heavy variable 5-17 (Fragment) | 12905 | 25 | 4 | 1 |
| A0A075B5Q2 | Immunoglobulin heavy variable 5-9 (Fragment) | 12795 | 25 | 4 | 1 |
| A0A075B5Q6 | Immunoglobulin heavy variable 5-9-1 | 13067 | 25 | 4 | 1 |
| A0A075B5T2 | Immunoglobulin heavy variable 6-3 (Fragment) | 13259 | 40 | 5 | 1 |
| A0A075B5T3 | Immunoglobulin heavy variable 6-6 (Fragment) | 13293 | 47 | 6 | 2 |
| A0A075B5R3 | Immunoglobulin heavy variable 7-2 | 13351 | 26 | 4 | 1 |
| A0A075B5R2 | Immunoglobulin heavy variable 7-3 (Fragment) | 13457 | 33 | 5 | 2 |
| A0A0A6YXQ0 | Immunoglobulin heavy variable 8-8 (Fragment) | 13185 | 11 | 2 | 1 |
| A0A075B5S9 | Immunoglobulin heavy variable 9-4 | 10934 | 31 | 3 | 3 |
| A0A0A6YWI9 | Immunoglobulin heavy variable V1-11 (Fragment) | 12865 | 17 | 2 | 2 |
| A0A0A6YXN4 | Immunoglobulin heavy variable V1-18 (Fragment) | 12913 | 56 | 5 | 3 |
| A0A075B5U6 | Immunoglobulin heavy variable V1-20 | 13071 | 35 | 4 | 2 |
| A0A075B5R9 | Immunoglobulin heavy variable V14-3 (Fragment) | 12975 | 27 | 3 | 1 |
| A0A075B5T5 | Immunoglobulin heavy variable V1-5 | 10846 | 19 | 1 | 1 |
| A0A075B5X2 | Immunoglobulin heavy variable V1-63 (Fragment) | 12900 | 27 | 2 | 1 |
| A0A075B5X6 | Immunoglobulin heavy variable V1-67 | 10976 | 32 | 3 | 3 |
| A0A075B5Y1 | Immunoglobulin heavy variable V1-74 | 12900 | 29 | 3 | 1 |
| A0A075B5X4 | Immunoglobulin heavy variable V8-11 (Fragment) | 13082 | 28 | 3 | 2 |
| A0A0G2JDE1 | Immunoglobulin heavy variable V8-12 (Fragment) | 13263 | 24 | 3 | 2 |
| A0A0A6YY60 | Immunoglobulin heavy variable V8-5 (Fragment) | 13172 | 20 | 2 | 1 |
| P01592 | Immunoglobulin J chain | 18014 | 40 | 7 | 7 |
| A0A140T8M9 | Immunoglobulin kappa chain variable 1-122 (Fragment) | 13117 | 17 | 2 | 1 |
| A0A075B5M8 | Immunoglobulin kappa chain variable 12-38 | 12428 | 54 | 4 | 4 |
| A0A140T8N3 | Immunoglobulin kappa chain variable 13-84 (Fragment) | 12617 | 37 | 4 | 2 |
| A0A075B666 | Immunoglobulin kappa chain variable 13-85 (Fragment) | 12709 | 37 | 3 | 1 |
| A0A075B5K7 | Immunoglobulin kappa chain variable 14-100 O | 12650 | 14 | 1 | 1 |
| A0A140T8P3 | Immunoglobulin kappa chain variable 15-103 (Fragment) | 12547 | 14 | 2 | 1 |
| A0A140T8N1 | Immunoglobulin kappa chain variable 1-88 (Fragment) | 13167 | 38 | 3 | 1 |
| A0A075B6D5 | Immunoglobulin kappa chain variable 19-93 | 12735 | 26 | 3 | 2 |
| A0A075B5M2 | Immunoglobulin kappa chain variable 4-61 | 10153 | 31 | 2 | 1 |
| A0A075B5L3 | Immunoglobulin kappa chain variable 4-90 (Fragment) | 12609 | 14 | 1 | 1 |
| A0A075B5L2 | Immunoglobulin kappa chain variable 4-91 (Fragment) | 12724 | 37 | 3 | 1 |
| A0A0B4J1J2 | Immunoglobulin kappa chain variable 5-43 (Fragment) | 12600 | 23 | 2 | 1 |
| A0A140T8P5 | Immunoglobulin kappa chain variable 8-24 (Fragment) | 13264 | 35 | 4 | 2 |
| A0A075B5N4 | Immunoglobulin kappa chain variable 8-27 | 10976 | 60 | 5 | 2 |
| A0A140T8M3 | Immunoglobulin kappa chain variable 8-30 (Fragment) | 13335 | 50 | 5 | 2 |
| A0A075B5K2 | Immunoglobulin kappa chain variable 9-124 | 10420 | 40 | 3 | 2 |
| P01837 | Immunoglobulin kappa constant | 11934 | 92 | 11 | 11 |
| A0A0B4J1I0 | Immunoglobulin kappa variable 1-110 (Fragment) | 13079 | 43 | 4 | 1 |
| A0A140T8M0 | Immunoglobulin kappa variable 1-117 (Fragment) | 13117 | 43 | 4 | 1 |
| A0A0B4J1H7 | Immunoglobulin kappa variable 1-135 (Fragment) | 13305 | 46 | 7 | 7 |
| A0A140T8M2 | Immunoglobulin kappa variable 12-44 (Fragment) | 12557 | 41 | 4 | 1 |
| A0A140T8P6 | Immunoglobulin kappa variable 12-46 (Fragment) | 12562 | 41 | 3 | 2 |
| A0A075B5K9 | Immunoglobulin kappa variable 12-98 | 12345 | 17 | 1 | 1 |
| A0A075B5K0 | Immunoglobulin kappa variable 14-126 (Fragment) | 13058 | 42 | 4 | 2 |
| A0A075B5J7 | Immunoglobulin kappa variable 14-130 | 13045 | 11 | 1 | 1 |
| A0A0B4J1I1 | Immunoglobulin kappa variable 16-104 (Fragment) | 12875 | 23 | 2 | 2 |
| A0A075B5K3 | Immunoglobulin kappa variable 17-121 | 12692 | 34 | 2 | 2 |
| A0A075B5P1 | Immunoglobulin kappa variable 3-1 (Fragment) | 12972 | 39 | 3 | 2 |
| A0A075B5N9 | Immunoglobulin kappa variable 3-7 | 10856 | 36 | 3 | 1 |
| A0A0B4J1J0 | Immunoglobulin kappa variable 4-50 (Fragment) | 12808 | 31 | 3 | 3 |
| A0A075B677 | Immunoglobulin kappa variable 4-53 | 10366 | 64 | 3 | 3 |
| A0A0B4J1I9 | Immunoglobulin kappa variable 4-55 (Fragment) | 12736 | 27 | 2 | 1 |
| A0A0A6YYE7 | Immunoglobulin kappa variable 4-57 (Fragment) | 12689 | 62 | 4 | 2 |
| A0A075B5M4 | Immunoglobulin kappa variable 4-57-1 (Fragment) | 12723 | 32 | 3 | 1 |
| A0A075B5M1 | Immunoglobulin kappa variable 4-63 | 10157 | 57 | 4 | 1 |
| A0A0B4J1I7 | Immunoglobulin kappa variable 4-68 (Fragment) | 12752 | 17 | 1 | 1 |
| A0A0B4J1I3 | Immunoglobulin kappa variable 4-74 (Fragment) | 12892 | 37 | 2 | 1 |
| A0A075B5L7 | Immunoglobulin kappa variable 4-80 (Fragment) | 12562 | 14 | 1 | 1 |
| A0A075B5M7 | Immunoglobulin kappa variable 5-39 | 10345 | 54 | 4 | 2 |
| A0A075B5N7 | Immunoglobulin kappa variable 6-13 | 10441 | 42 | 5 | 2 |
| A0A140T8P1 | Immunoglobulin kappa variable 6-14 (Fragment) | 12848 | 40 | 5 | 2 |
| A0A140T8M5 | Immunoglobulin kappa variable 6-15 (Fragment) | 12757 | 45 | 5 | 2 |
| A0A140T8P2 | Immunoglobulin kappa variable 6-20 (Fragment) | 12640 | 32 | 3 | 1 |
| A0A140T8N5 | Immunoglobulin kappa variable 6-23 (Fragment) | 12787 | 45 | 5 | 1 |
| A0A140T8N9 | Immunoglobulin kappa variable 6-32 (Fragment) | 12656 | 45 | 5 | 3 |
| A0A140T8M4 | Immunoglobulin kappa variable 8-19 | 11116 | 45 | 4 | 1 |
| A0A140T8P7 | Immunoglobulin kappa variable 8-21 (Fragment) | 13128 | 32 | 4 | 1 |
| A0A075B5N5 | Immunoglobulin kappa variable 8-26 (Fragment) | 13821 | 12 | 2 | 1 |
| A0A075B5N3 | Immunoglobulin kappa variable 8-28 | 10903 | 59 | 5 | 3 |
| A0A140T8N8 | Immunoglobulin kappa variable 9-123 (Fragment) | 12815 | 32 | 3 | 2 |
| A0A075B664 | Immunoglobulin lambda variable 2 | 12165 | 42 | 3 | 2 |
| A0A0B4J1K5 | Immunoglobulin lambda variable 3 (Fragment) | 13416 | 39 | 3 | 3 |
| Q9DBD0 | Inhibitor of carbonic anhydrase | 76766 | 63 | 36 | 34 |
| P70389 | Insulin-like growth factor-binding protein complex acid labile subunit | 66960 | 29 | 14 | 14 |
| E9PVD2 | Inter alpha-trypsin inhibitor heavy chain 4 | 104588 | 45 | 34 | 1 |
| A6X935 | Inter alpha-trypsin inhibitor heavy chain 4 | 104660 | 45 | 34 | 1 |
| Q61702 | Inter-alpha-trypsin inhibitor heavy chain H1 | 101067 | 31 | 22 | 21 |
| Q61703 | Inter-alpha-trypsin inhibitor heavy chain H2 | 105928 | 34 | 26 | 26 |
| Q61704 | Inter-alpha-trypsin inhibitor heavy chain H3 | 99358 | 37 | 24 | 23 |
| O35664 | Interferon alpha/beta receptor 2 | 56578 | 4 | 2 | 2 |
| Q61730 | Interleukin-1 receptor accessory protein | 65741 | 18 | 9 | 9 |
| Q8K057 | Intraflagellar transport protein 80 homolog | 87811 | 1 | 1 | 1 |
| G5E8P9 | Katanin p60 ATPase-containing subunit A-like 1 (Fragment) | 26782 | 3 | 1 | 1 |
| A2A513 | Keratin type I cytoskeletal 10 | 57041 | 14 | 10 | 6 |
| Q61781 | Keratin type I cytoskeletal 14 | 52867 | 29 | 15 | 1 |
| Q61414 | Keratin type I cytoskeletal 15 | 49138 | 12 | 9 | 1 |
| Q9Z2K1 | Keratin type I cytoskeletal 16 | 51606 | 17 | 9 | 3 |
| Q9QWL7 | Keratin type I cytoskeletal 17 | 48162 | 52 | 25 | 11 |
| P19001 | Keratin type I cytoskeletal 19 | 44542 | 15 | 9 | 2 |
| Q6IFX2 | Keratin type I cytoskeletal 42 | 50133 | 33 | 16 | 6 |
| Q99M73 | Keratin type II cuticular Hb4 | 64983 | 6 | 5 | 1 |
| P04104 | Keratin type II cytoskeletal 1 | 65606 | 9 | 9 | 3 |
| Q3TTY5 | Keratin type II cytoskeletal 2 epidermal | 70923 | 13 | 14 | 7 |
| Q3UV17 | Keratin type II cytoskeletal 2 oral | 62845 | 11 | 10 | 1 |
| Q922U2 | Keratin type II cytoskeletal 5 | 61767 | 27 | 25 | 10 |
| P50446 | Keratin type II cytoskeletal 6A | 59335 | 30 | 25 | 7 |
| Q6IME9 | Keratin type II cytoskeletal 72 | 56750 | 8 | 5 | 1 |
| Q6NXH9 | Keratin type II cytoskeletal 73 | 58911 | 12 | 9 | 1 |
| Q8BGZ7 | Keratin type II cytoskeletal 75 | 59741 | 21 | 16 | 1 |
| Q8VED5 | Keratin type II cytoskeletal 79 | 57552 | 13 | 12 | 2 |
| Q3TBQ1 | Keratin type II cytoskeletal 80 | 24669 | 4 | 1 | 1 |
| E9Q0F0 | Keratin 78 | 112265 | 2 | 3 | 2 |
| G5E8B6 | Kin of IRRE-like protein 3 | 80405 | 1 | 1 | 1 |
| A0A087WS04 | Kinectin | 140547 | 1 | 1 | 1 |
| A0A338P699 | Kininogen 2 (Fragment) | 15324 | 56 | 9 | 1 |
| A0A0R4J038 | Kininogen-1 | 73101 | 46 | 28 | 23 |
| B1B1E2 | Latent-transforming growth factor beta-binding protein 1 (Fragment) | 107029 | 2 | 1 | 1 |
| Q91XL1 | Leucine-rich HEV glycoprotein | 37431 | 26 | 9 | 8 |
| E9PVE2 | Leucine-rich repeat-containing protein 45 | 47351 | 3 | 2 | 1 |
| P42703 | Leukemia inhibitory factor receptor | 122574 | 21 | 20 | 20 |
| A2AC65 | Lipopolysaccharide-binding protein | 13134 | 9 | 1 | 1 |
| Q8CGK3 | Lon protease homolog mitochondrial | 105843 | 1 | 1 | 1 |
| P51885 | Lumican | 38265 | 43 | 13 | 13 |
| P17897 | Lysozyme C-1 | 16794 | 8 | 1 | 1 |
| P09581 | Macrophage colony-stimulating factor 1 receptor | 109179 | 9 | 10 | 10 |
| P11589 | Major urinary protein 2 | 20664 | 47 | 7 | 7 |
| A0A571BF69 | Maltase-glucoamylase | 413024 | 3 | 9 | 9 |
| P98064 | Mannan-binding lectin serine protease 1 | 79968 | 16 | 9 | 9 |
| P39039 | Mannose-binding protein A | 25396 | 17 | 4 | 4 |
| P41317 | Mannose-binding protein C | 25957 | 39 | 7 | 7 |
| P11034 | Mast cell protease 1 | 27013 | 30 | 5 | 4 |
| A0A087WS76 | Mitogen-activated protein kinase kinase kinase 19 (Fragment) | 135992 | 1 | 1 | 1 |
| Q61083 | Mitogen-activated protein kinase kinase kinase 2 | 69574 | 2 | 2 | 1 |
| P28665 | Murinoglobulin-1 | 165297 | 61 | 84 | 52 |
| P28666 | Murinoglobulin-2 | 162381 | 22 | 32 | 1 |
| A0A140LIG1 | N-acetylglucosaminyl-phosphatidylinositol biosynthetic protein (Fragment) | 27766 | 3 | 1 | 1 |
| Q8VCS0 | N-acetylmuramoyl-L-alanine amidase | 57707 | 13 | 6 | 6 |
| A1Z198 | NACHT LRR and PYD domains-containing protein 1b allele 2 | 134163 | 1 | 1 | 1 |
| Q6P5U7 | NACHT and WD repeat domain-containing protein 2 | 197413 | 1 | 2 | 1 |
| E9Q1W3 | Nebulin | 828679 | 0 | 2 | 2 |
| P54729 | NEDD8 ultimate buster 1 | 70307 | 1 | 1 | 1 |
| Q6ZWR6 | Nesprin-1 | 1009946 | 0 | 1 | 1 |
| P10493 | Nidogen-1 | 136538 | 1 | 1 | 1 |
| Q9R0G8 | Nik-related protein kinase | 163647 | 1 | 1 | 1 |
| Q63850 | Nuclear pore glycoprotein p62 | 53255 | 1 | 1 | 1 |
| Q571H0 | Nucleolar pre-ribosomal-associated protein 1 | 254611 | 0 | 1 | 1 |
| Q9D3H2 | Odorant-binding protein 1a | 18469 | 50 | 9 | 5 |
| A2AEP0 | Odorant-binding protein 1b | 19394 | 70 | 13 | 12 |
| Q9EPF6 | Olfactory receptor 704 | 35120 | 2 | 1 | 1 |
| Q3UU35 | Ovostatin homolog | 162340 | 1 | 1 | 1 |
| Q9ET66 | Peptidase inhibitor 16 | 53650 | 3 | 1 | 1 |
| V9GX31 | Peptidyl-prolyl cis-trans isomerase | 8561 | 12 | 1 | 1 |
| Q62009 | Periostin | 93144 | 8 | 4 | 4 |
| P16301 | Phosphatidylcholine-sterol acyltransferase | 49747 | 11 | 5 | 5 |
| O70362 | Phosphatidylinositol-glycan-specific phospholipase D | 93255 | 22 | 15 | 15 |
| P55065 | Phospholipid transfer protein | 54453 | 14 | 6 | 5 |
| Q6PAC4 | Photoreceptor cilium actin regulator | 139297 | 1 | 1 | 1 |
| P97298 | Pigment epithelium-derived factor | 46234 | 17 | 6 | 6 |
| P26262 | Plasma kallikrein | 71383 | 40 | 24 | 24 |
| P97290 | Plasma protease C1 inhibitor | 55585 | 38 | 15 | 15 |
| P20918 | Plasminogen | 90808 | 70 | 50 | 49 |
| Q9Z126 | Platelet factor 4 | 11243 | 29 | 3 | 3 |
| Q60963 | Platelet-activating factor acetylhydrolase | 49258 | 24 | 10 | 10 |
| Q9QXS1 | Plectin | 534193 | 0 | 1 | 1 |
| O70570 | Polymeric immunoglobulin receptor | 84999 | 13 | 9 | 9 |
| P0CG50 | Polyubiquitin-C | 82550 | 3 | 2 | 2 |
| Q5SW25 | POM121-like protein 2 | 103220 | 1 | 1 | 1 |
| A0A338P719 | Predicted gene 1043 | 157157 | 0 | 1 | 1 |
| B8JJN0 | Predicted gene 20547 | 142325 | 28 | 38 | 38 |
| A0A0G2JGT0 | Predicted gene 43218 (Fragment) | 12726 | 17 | 2 | 1 |
| E9PUM5 | Predicted gene 4788 | 91445 | 9 | 10 | 3 |
| A2AEN9 | Predicted gene 5938 | 18508 | 50 | 8 | 5 |
| A0A2R8VHP3 | Predicted pseudogene 5478 | 57920 | 10 | 7 | 1 |
| Q61838 | Pregnancy zone protein | 165852 | 67 | 84 | 82 |
| P48678 | Prelamin-A/C | 74238 | 2 | 1 | 1 |
| A2AKX3 | Probable helicase senataxin | 297588 | 0 | 1 | 1 |
| P28798 | Progranulin | 63458 | 5 | 2 | 2 |
| P02816 | Prolactin-inducible protein homolog | 16823 | 5 | 1 | 1 |
| Q91ZX7 | Prolow-density lipoprotein receptor-related protein 1 | 504745 | 0 | 2 | 1 |
| P11680 | Properdin | 50327 | 21 | 8 | 7 |
| K3W4L3 | Prosaposin | 61292 | 5 | 2 | 2 |
| Q9Z1R9 | Protease serine 1 (trypsin 1) | 26135 | 8 | 1 | 1 |
| Q07456 | Protein AMBP | 39029 | 29 | 9 | 9 |
| Q924A2 | Protein capicua homolog | 258127 | 0 | 1 | 1 |
| Q6VGS5 | Protein Daple | 226533 | 0 | 1 | 1 |
| Q8CAS9 | Protein mono-ADP-ribosyltransferase PARP9 | 96659 | 1 | 1 | 1 |
| Q5SW75 | Protein phosphatase Slingshot homolog 2 | 158230 | 0 | 1 | 1 |
| Q8R121 | Protein Z-dependent protease inhibitor | 51797 | 28 | 9 | 8 |
| P19221 | Prothrombin | 70269 | 53 | 35 | 33 |
| Q922P9 | Putative oxidoreductase GLYR1 | 59716 | 1 | 1 | 1 |
| P52480 | Pyruvate kinase PKM | 57845 | 2 | 1 | 1 |
| G3X9J4 | RAB11-binding protein RELCH | 134581 | 1 | 1 | 1 |
| P70392 | Ras-specific guanine nucleotide-releasing factor 2 | 135667 | 1 | 1 | 1 |
| Q00724 | Retinol-binding protein 4 | 23206 | 48 | 9 | 9 |
| S4R1K5 | Rho guanine nucleotide exchange factor 9 | 49184 | 2 | 1 | 1 |
| A2AVJ7 | Ribosome-binding protein 1 | 158395 | 1 | 1 | 1 |
| Q9CPN9 | RIKEN cDNA 2210010C04 gene | 26422 | 3 | 1 | 1 |
| E9PVG8 | RIKEN cDNA 9530053A07 gene | 280230 | 0 | 1 | 1 |
| H3BL88 | RIKEN cDNA 9930021J03 gene (Fragment) | 213683 | 1 | 2 | 1 |
| Q0VBL3 | RNA-binding protein 15 | 105722 | 1 | 1 | 1 |
| E9Q401 | Ryanodine receptor 2 | 564827 | 1 | 2 | 2 |
| A0A140LJF7 | Ryanodine receptor 3 | 550840 | 0 | 1 | 1 |
| Q8K1I3 | Secreted phosphoprotein 24 | 23136 | 11 | 2 | 2 |
| G5E8B4 | Secretoglobin family 2B member 2 | 12956 | 39 | 4 | 4 |
| P70274 | Selenoprotein P | 42706 | 21 | 8 | 7 |
| A0A0R4J0I1 | Serine protease inhibitor A3K | 46673 | 40 | 17 | 1 |
| P07759 | Serine protease inhibitor A3K | 46880 | 56 | 26 | 6 |
| Q03734 | Serine protease inhibitor A3M | 47064 | 43 | 18 | 6 |
| Q91WP6 | Serine protease inhibitor A3N | 46718 | 51 | 21 | 14 |
| A0A087WPS9 | Serine/arginine repetitive matrix protein 2 (Fragment) | 9822 | 14 | 1 | 1 |
| Q921I1 | Serotransferrin | 76724 | 77 | 66 | 63 |
| P07724 | Serum albumin | 68693 | 90 | 69 | 69 |
| P05366 | Serum amyloid A-1 protein | 13770 | 25 | 3 | 3 |
| P31532 | Serum amyloid A-4 protein | 15088 | 42 | 5 | 5 |
| P12246 | Serum amyloid P-component | 26247 | 49 | 13 | 13 |
| P52430 | Serum paraoxonase/arylesterase 1 | 39565 | 49 | 13 | 12 |
| G5E8D1 | Small proline-rich protein 2K | 7559 | 13 | 1 | 1 |
| Q64442 | Sorbitol dehydrogenase | 38249 | 7 | 2 | 2 |
| Q2TBA9 | Sox5 protein | 79257 | 1 | 1 | 1 |
| P70663 | SPARC-like protein 1 | 72287 | 2 | 1 | 1 |
| Q6P5D8 | Structural maintenance of chromosomes flexible hinge domain-containing protein 1 | 225646 | 0 | 1 | 1 |
| Q8BND5 | Sulfhydryl oxidase 1 | 82785 | 32 | 20 | 20 |
| G3UW60 | Sushi domain-containing 5 | 66803 | 1 | 1 | 1 |
| Q8CHC4 | Synaptojanin-1 | 172616 | 1 | 1 | 1 |
| Q8R570 | Synaptosomal-associated protein 47 | 46524 | 2 | 1 | 1 |
| A0A5F8MPX2 | Synaptotagmin-like protein 2 | 233242 | 0 | 1 | 1 |
| D6RFB9 | Syntaxin-1A | 29485 | 4 | 1 | 1 |
| E9PUM4 | Talin-2 | 271665 | 0 | 1 | 1 |
| Q80YX0 | Tenascin | 91210 | 1 | 1 | 1 |
| F8VPK0 | Tetratricopeptide repeat domain 37 | 173941 | 0 | 1 | 1 |
| E9Q6P5 | Tetratricopeptide repeat protein 7B | 94203 | 1 | 1 | 1 |
| Q6P4U0 | Thrombospondin type-1 domain-containing protein 7B | 179308 | 1 | 1 | 1 |
| F7CR78 | Titin (Fragment) | 914024 | 0 | 1 | 1 |
| O88968 | Transcobalamin-2 | 47586 | 11 | 3 | 3 |
| Q62351 | Transferrin receptor protein 1 | 85731 | 13 | 7 | 7 |
| P07309 | Transthyretin | 15776 | 67 | 7 | 7 |
| Q8BQ33 | Treslin | 208333 | 1 | 1 | 1 |
| Q8C013 | Trophoblast glycoprotein-like | 41085 | 2 | 1 | 1 |
| Q792Z1 | Trypsin 10 | 26221 | 8 | 1 | 1 |
| Q80WC1 | Ubinuclein-2 | 141740 | 1 | 2 | 1 |
| Q9ES00 | Ubiquitin conjugation factor E4 B | 133317 | 1 | 1 | 1 |
| E9Q6R7 | Utrophin | 392707 | 0 | 1 | 1 |
| Q8CGB3 | Uveal autoantigen with coiled-coil domains and ankyrin repeats | 160812 | 0 | 1 | 1 |
| P29533 | Vascular cell adhesion protein 1 | 81317 | 2 | 1 | 1 |
| Q9QZ25 | Vascular non-inflammatory molecule 3 | 56305 | 10 | 4 | 4 |
| P21614 | Vitamin D-binding protein | 53600 | 75 | 33 | 33 |
| Q9CQW3 | Vitamin K-dependent protein Z | 44304 | 9 | 3 | 3 |
| P29788 | Vitronectin | 54849 | 42 | 17 | 17 |
| A0A3B2W4A7 | Vomeronasal 2 receptor 53 | 96810 | 1 | 1 | 1 |
| Q8C2E7 | WASH complex subunit 5 | 134110 | 1 | 1 | 1 |
| A2A485 | Zinc finger MYND-type-containing 8 | 120841 | 1 | 1 | 1 |
| Q9JJN2 | Zinc finger homeobox protein 4 | 392325 | 0 | 1 | 1 |
| A0A087WSF4 | Zinc finger protein 976 | 75905 | 2 | 1 | 1 |
| Q64726 | Zinc-alpha-2-glycoprotein | 35332 | 43 | 15 | 15 |

**Supplementary Table 2.** Differentially abundant proteins on the serum proteome during *S. mansoni* infection.

| **Accession** | **Description** | **Abbreviation** | **Coverage (%)** | **Molecular Mass (Da)** | **Log2 Fold-change 5th week post Infection/Control** | **Log2 Fold-change 7th week post Infection/Control** | **Significance** | **Sample Profile Ratio**  **(Control:5^th^ Week:7^th^ Week)** |
| --- | --- | --- | --- | --- | --- | --- | --- | --- |
|  | **Lipid Metabolism and Transport Proteins** |  |  |  |  |  |  |  |
| Q60994 | Adiponectin | Adipoq | 13 | 26809 | -1,18 | -2,03 | 29,32 | 1.00:0.44:0.24 |
| P09813 | Apolipoprotein A-II | Apoa2 | 15 | 11309 | 0,06 | -1,09 | 23,74 | 1.00:1.04:0.47 |
| P06728 | Apolipoprotein A-IV | Apoa4 | 84 | 45029 | -0,49 | 1,55 | 82,04 | 1.00:0.71:2.92 |
| E9Q1Y3 | Apolipoprotein B-100 (Fragment) | Apoab | 10 | 503910 | 0,35 | 2,32 | 59,49 | 1.00:1.27:4.99 |
| Q05020 | Apolipoprotein C-II | Apoc2 | 33 | 10741 | -4,74 | 0,07 | 55,88 | 1.00:0.04:1.05 |
| P33622 | Apolipoprotein C-III | Apoc3 | 46 | 10982 | -1,03 | 0,99 | 74,23 | 1.00:0.49:1.99 |
| Q61268 | Apolipoprotein C-IV | Apoc4 | 16 | 14288 | -0,71 | 1,11 | 36,26 | 1.00:0.61:2.16 |
| P51910 | Apolipoprotein D | Apod | 31 | 21530 | -0,21 | 1,35 | 33,08 | 1.00:0.86:2.54 |
| Q9QWK4 | CD5 antigen-like | Cd5l | 42 | 38863 | 1,17 | 2,14 | 30,03 | 1.00:2.24:4.40 |
| Q06770 | Corticosteroid-binding globulin | Serpina6 | 28 | 44769 | -0,02 | -1,90 | 63,07 | 1.00:0.98:0.27 |
| Q60963 | Platelet-activating factor acetylhydrolase | Pla2gt | 10 | 49258 | 1,11 | 3,36 | 67,75 | 1.00:2.16:10.30 |
| P16301 | Phosphatidylcholine-sterol acyltransferase | Lcat | 8 | 49747 | 0,60 | 2,76 | 64,11 | 1.00:1.51:6.76 |
| A2A5K2 | Phospholipid transfer protein | Pltp | 9 | 49023 | 0,63 | 1,50 | 19,34 | 1.00:1.55:2.82 |
|  | **Acute Phase Response/Inflammation Proteins** |  |  |  |  |  |  |  |
| Q00897 | Alpha-1-antitrypsin 1-4 | Serpina1d | 45 | 45998 | 2,15 | 5,03 | 124,24 | 1.00:4.44:32.63 |
| Q60590 | Alpha-1-acid glycoprotein 1 | Orm1 | 28 | 23895 | 0,54 | 2,08 | 37,12 | 1.00:1.46:4.24 |
| P29699 | Alpha-2-HS-glycoprotein | Ahsg | 53 | 37326 | -0,24 | 1,27 | 15,94 | 1.00:0.85:2.41 |
| P14847 | C-reactive protein | Crp | 10 | 25360 | 0,33 | 1,54 | 22,05 | 1.00:1.25:2.91 |
| B7ZNJ1 | Fibronectin | Fn1 | 45 | 239720 | 0,43 | 2,41 | 65,17 | 1.00:1.35:5.30 |
| Q6S9I0 | Kininogen 2 | Kng2 | 30 | 47887 | 1,73 | 2,40 | 41,95 | 1.00:3.32:5.30 |
| P12246 | Serum amyloid P-component | Apcs | 45 | 26247 | 0,82 | 2,77 | 91,06 | 1.00:1.77:6.84 |
|  | **Immune Response Proteins** |  |  |  |  |  |  |  |
| Q8HWB2 | Histocompatibility 2 Q region locus 4 | - | 15 | 39618 | 1,28 | 3,33 | 42,27 | 1.00:2.42:10.08 |
| P01878 | Ig alpha chain C region | - | 42 | 36876 | -1,00 | 2,04 | 132,42 | 1.00:2.42:10.08 |
| A0A075B5P4 | Ig gamma-1 chain C region secreted form (Fragment) | - | 52 | 35752 | 4,27 | 4,90 | 119,12 | 1.00:0.50:4.09 |
| P01868 | Ig gamma-1 chain C region secreted form | - | 56 | 35705 | 6,01 | 7,30 | 71,99 | 1.00:19.26:29.96 |
| P01867 | Ig gamma-2B chain C region | - | 35 | 44259 | -0,18 | 1,11 | 17,45 | 1.00:64.31:158.27 |
| P01806 | Ig heavy chain V region 441 | - | 72 | 12911 | 1,27 | 5,37 | 200 | 1.00:0.88:2.16 |
| P01790 | Ig heavy chain V region M511 | - | 52 | 13652 | 1,20 | 2,62 | 54,01 | 1.00:2.42:41.19 |
| P01820 | Ig heavy chain V region PJ14 | - | 10 | 12447 | 2,36 | 0,73 | 16,68 | 1.00:2.30:6.14 |
| P01801 | Ig heavy chain V-III region J606 | - | 43 | 12810 | 1,32 | 3,40 | 64,4 | 1.00:5.15:1.66 |
| P03976 | Ig kappa chain V-II region 17S29.1 | - | 27 | 12390 | 3,64 | 7,77 | 116,64 | 1.00:2.50:10.52 |
| P01629 | Ig kappa chain V-II region 2S1.3 | - | 45 | 12221 | 1,09 | 6,06 | 107,66 | 1.00:12.42:218.69 |
| P01630 | Ig kappa chain V-II region 7S34.1 | - | 27 | 12496 | 1,51 | 3,54 | 14,9 | 1.00:2.12:66.86 |
| P01662 | Ig kappa chain V-III region ABPC 22/PC 9245 | - | 65 | 12041 | 5,70 | 7,19 | 63,35 | 1.00:2.85:11.67 |
| P01656 | Ig kappa chain V-III region MOPC 70 | - | 46 | 11904 | 3,34 | 1,92 | 22,89 | 1.00:51.91:146.54 |
| P01668 | Ig kappa chain V-III region PC 7210 | - | 36 | 11950 | 2,45 | 3,09 | 57,42 | 1.00:10.12:3.80 |
| P01644 | Ig kappa chain V-V region HP R16.7 | - | 63 | 11910 | 2,22 | 3,50 | 80,6 | 1.00:5.45:8.51 |
| P01635 | Ig kappa chain V-V region K2 (Fragment) | - | 45 | 12581 | 4,53 | 5,97 | 59,61 | 1.00:4.66:11.32 |
| P01638 | Ig kappa chain V-V region L6 (Fragment) | - | 28 | 12986 | 4,49 | 4,33 | 62,49 | 1.00:23.01:62.54 |
| P01642 | Ig kappa chain V-V region L7 (Fragment) | - | 29 | 12615 | 3,42 | 3,80 | 53,41 | 1.00:22.56:20.14 |
| P01843 | Ig lambda-1 chain C region | - | 81 | 11575 | 1,14 | 3,23 | 82,15 | 1.00:10.76:13.95 |
| P01723 | Ig lambda-1 chain V region | - | 56 | 12222 | 1,56 | 2,96 | 16,55 | 1.00:2.21:9.38 |
| P01844 | Ig lambda-2 chain C region | - | 71 | 11255 | 2,79 | 3,78 | 60,94 | 1.00:2.95:7.79 |
| A0A075B5P5 | Immunoglobulin heavy constant gamma 3 (Fragment) | - | 51 | 36317 | 0,24 | 1,31 | 23,11 | 1.00:6.92:13.80 |
| P01872 | Immunoglobulin heavy constant mu | - | 58 | 49972 | 0,50 | 3,26 | 200 | 1.00:1.19:2.48 |
| A0A075B5R6 | Immunoglobulin heavy variable 11-1 (Fragment) | - | 28 | 13208 | 0,18 | 2,95 | 52,96 | 1.00:1.41:9.60 |
| A0A075B5Q9 | Immunoglobulin heavy variable 5-15 | - | 33 | 12943 | 4,82 | 4,78 | 23,25 | 1.00:1.13:7.76 |
| A0A075B5S9 | Immunoglobulin heavy variable 9-4 | - | 31 | 10934 | 3,07 | 2,98 | 57,4 | 1.00:28.30:27.47 |
| A0A075B5T5 | Immunoglobulin heavy variable V1-5 | - | 19 | 10846 | 3,41 | 3,33 | 71,78 | 1.00:8.39:7.87 |
| A0A075B5X6 | Immunoglobulin heavy variable V1-67 | - | 11 | 10976 | 2,99 | 3,43 | 25,49 | 1.00:10.63:10.05 |
| A0A075B5X4 | Immunoglobulin heavy variable V8-11 (Fragment) | - | 22 | 13082 | 3,94 | 4,19 | 27,06 | 1.00:8.01:10.81 |
| P01592 | Immunoglobulin J chain | - | 28 | 18014 | 0,62 | 3,46 | 91,23 | 1.00:15.31:18.20 |
| A0A140T8P3 | Immunoglobulin kappa chain variable 15-103 (Fragment) | - | 14 | 12547 | -2,18 | 2,61 | 24,33 | 1.00:1.54:11.02 |
| A0A075B6D5 | Immunoglobulin kappa chain variable 19-93 | - | 19 | 12735 | 3,74 | 5,16 | 63 | 1.00:0.22:6.10 |
| A0A140T8P5 | Immunoglobulin kappa chain variable 8-24 (Fragment) | - | 35 | 13264 | 1,30 | 2,93 | 62,04 | 1.00:13.39:35.89 |
| A0A075B5N4 | Immunoglobulin kappa chain variable 8-27 | - | 60 | 10976 | 0,72 | 5,40 | 143,31 | 1.00:2.46:7.60 |
| A0A140T8M3 | Immunoglobulin kappa chain variable 8-30 (Fragment) | - | 35 | 13335 | 2,35 | 3,25 | 17,51 | 1.00:1.65:42.27 |
| A0A140T8N0 | Immunoglobulin kappa chain variable 9-120 (Fragment) | - | 51 | 12805 | 1,31 | 3,15 | 79,21 | 1.00:5.11:9.56 |
| P01837 | Immunoglobulin kappa constant | - | 87 | 11934 | 1,82 | 3,10 | 121,73 | 1.00:2.48:8.91 |
| A0A0B4J1H7 | Immunoglobulin kappa variable 1-135 (Fragment) | - | 46 | 13305 | 4,10 | 3,57 | 66,03 | 1.00:3.53:8.60 |
| A0A140T8P6 | Immunoglobulin kappa variable 12-46 (Fragment) | - | 45 | 12562 | 6,35 | 6,71 | 64,15 | 1.00:17.09:11.82 |
| A0A0B4J1I1 | Immunoglobulin kappa variable 16-104 (Fragment) | - | 23 | 12875 | 3,14 | 3,78 | 65,41 | 1.00:81.29:104.86 |
| A0A075B5J9 | Immunoglobulin kappa variable 17-127 | - | 14 | 12330 | 3,95 | 4,22 | 29,24 | 1.00:8.79:13.70 |
| A0A075B5N9 | Immunoglobulin kappa variable 3-7 | - | 36 | 10856 | 2,87 | 3,15 | 25,25 | 1.00:15.47:18.61 |
| A0A0B4J1I9 | Immunoglobulin kappa variable 4-55 (Fragment) | - | 43 | 12736 | 2,71 | 2,62 | 24,79 | 1.00:7.31:8.87 |
| A0A0B4J1I7 | Immunoglobulin kappa variable 4-68 (Fragment) | - | 17 | 12752 | 4,14 | 4,84 | 23,36 | 1.00:6.54:6.14 |
| A0A075B5M7 | Immunoglobulin kappa variable 5-39 | - | 47 | 10345 | 2,48 | 4,30 | 64,49 | 1.00:17.69:28.69 |
| A0A140T8M5 | Immunoglobulin kappa variable 6-15 (Fragment) | - | 53 | 12757 | 4,19 | 4,92 | 73,23 | 1.00:5.57:19.67 |
| A0A140T8Q3 | Immunoglobulin kappa variable 6-17 (Fragment) | - | 45 | 12760 | 3,16 | 3,49 | 26,58 | 1.00:18.26:30.17 |
| A0A140T8N9 | Immunoglobulin kappa variable 6-32 (Fragment) | - | 45 | 12656 | 3,30 | 4,82 | 71,54 | 1.00:8.92:11.26 |
| A0A075B5N3 | Immunoglobulin kappa variable 8-28 | - | 42 | 10903 | 5,23 | 3,57 | 47,46 | 1.00:9.86:28.24 |
| A0A075B664 | Immunoglobulin lambda variable 2 | - | 14 | 12165 | 2,22 | 4,11 | 14,21 | 1.00:37.53:11.84 |
| Q61730 | Interleukin-1 receptor accessory protein | - | 13 | 65741 | 0,09 | -1,96 | 64,2 | 1.00:1.07:0.26 |
| P09581 | Macrophage colony-stimulating factor 1 receptor | Csf1r | 5 | 109179 | 1,44 | 1,35 | 16,46 | 1.00:2.72:2.55 |
| Q8BND5 | Sulfhydryl oxidase 1 | - | 25 | 82785 | 1,30 | 1,52 | 20,33 | 1.00:2.47:2.88 |
|  | **Complement System Proteins** |  |  |  |  |  |  |  |
| P01027 | Complement C3 | C3 | 70 | 186483 | -0,40 | 1,72 | 84,32 | 1.00:0.76:3.29 |
| D3YXF5 | Complement component 7 | C7 | 6 | 93338 | -1,19 | 3,67 | 96,63 | 1.00:0.44:12.71 |
| A2A998 | Complement component C8 alpha chain | C8a | 9 | 61008 | 0,83 | -1,26 | 61,89 | 1.00:1.77:0.42 |
| P06683 | Complement component C9 | C | 10 | 62002 | 2,63 | 2,51 | 32,24 | 1.00:6.19:5.69 |
| A0A0A6YVP8 | Complement factor H | - | 59 | 80361 | 0,32 | 1,37 | 18,75 | 1.00:1.24:2.59 |
| P08607 | C4b-binding protein | C4b | 22 | 51524 | 0,55 | 1,67 | 26,04 | 1.00:1.46:3.18 |
| P11680 | Properdin | Cfp | 14 | 50327 | -0,42 | 1,92 | 86,36 | 1.00:0.75:3.78 |
|  | **Metal Binding** |  |  |  |  |  |  |  |
| G3X9T8 | Ceruloplasmin | Cp | 49 | 121080 | 0,97 | 1,64 | 19,67 | 1.00:1.96:3.14 |
| P01942 | Hemoglobin subunit alpha | Hba-a2 | 61 | 15085 | -1,03 | -2,31 | 50,95 | 1.00:0.49:0.20 |
| P02088 | Hemoglobin subunit beta-1 | Hba-bs | 84 | 15840 | -0,62 | -1,92 | 39,77 | 1.00:0.65:0.27 |
| P02089 | Hemoglobin subunit beta-2 | - | 80 | 15878 | -0,93 | -2,19 | 41,3 | 1.00:0.53:0.22 |
| Q91X72 | Hemopexin | Hpx | 60 | 51318 | 1,15 | 1,50 | 33,69 | 1.00:2.21:2.81 |
| Q07456 | Protein AMBP | Ambp | 22 | 39029 | -0,26 | 1,17 | 45,97 | 1.00:0.83:2.25 |
| Q921I1 | Serotransferrin | Trf | 74 | 76724 | 0,20 | 1,18 | 28,33 | 1.00:1.15:2.26 |
|  | **Hemostasis** |  |  |  |  |  |  |  |
| Q07968 | Coagulation factor XIII B chain | F13b | 5 | 76195 | -0,20 | -1,71 | 16,56 | 1.00:0.87:0.30 |
| E9PV24 | Fibrinogen alpha chain | Fga | 44 | 87429 | 0,19 | 1,60 | 51,35 | 1.00:1.15:3.03 |
| Q8K0E8 | Fibrinogen beta chain | Fgb | 77 | 54753 | 0,17 | 1,57 | 41,61 | 1.00:1.13:2.97 |
| Q8VCM7 | Fibrinogen gamma chain | Fgg | 73 | 49391 | 0,35 | 1,27 | 23,31 | 1.00:1.27:2.41 |
| O35930 | Platelet glycoprotein Ib alpha chain | Gp1ba | 4 | 80055 | -1,35 | -2,39 | 29,78 | 1.00:0.39:0.19 |
|  | **Proteolysis/Inhibition** |  |  |  |  |  |  |  |
| Q9JHH6 | Carboxypeptidase B2 | Cpb2 | 5 | 48871 | 0,06 | 1,77 | 45,5 | 1.00:1.04:3.42 |
| Q61704 | Inter-alpha-trypsin inhibitor heavy chain H3 | Serpina10 | 24 | 99358 | 0,27 | 2,06 | 42,84 | 1.00:1.21:4.17 |
| Q8R121 | Protein Z-dependent protease inhibitor | Itih3 | 12 | 51797 | 0,78 | 1,36 | 16,12 | 1.00:1.72:2.57 |
| Q91WP6 | Serine protease inhibitor A3N | Serpina3n | 43 | 46718 | 0,68 | 2,50 | 54,96 | 1.00:1.60:5.64 |
|  | **Proteins related to other processes** |  |  |  |  |  |  |  |
| Q3UTR7 | Angiotensin 1-10 | Agt | 14 | 52670 | 0,16 | 1,21 | 14,55 | 1.00:1.12:2.32 |
| Q9QXC1 | Fetuin-B | Fetub | 44 | 42713 | 0,69 | 1,08 | 15,99 | 1.00:1.62:2.12 |
| P70389 | Insulin-like growth factor-binding protein complex acid labile subunit | Igfals | 11 | 66960 | 0,27 | -1,42 | 39,96 | 1.00:1.20:0.37 |
| Q922U2 | Keratin type II cytoskeletal 5 | Krt5 | 21 | 61767 | -1,60 | -1,51 | 19,45 | 1.00:0.33:0.35 |
| P50446 | Keratin type II cytoskeletal 6A | Krt6a | 27 | 59335 | -4,07 | -3,52 | 63,03 | 1.00:0.06:0.09 |
| Q8VED5 | Keratin type II cytoskeletal 79 | - | 12 | 57552 | -1,82 | -0,79 | 19,24 | 1.00:0.28:0.58 |
| Q91XL1 | Leucine-rich HEV glycoprotein | Lrg1 | 24 | 37431 | 0,69 | 3,20 | 63,36 | 1.00:1.61:9.15 |
| P42703 | Leukemia inhibitory factor receptor | Lifr | 12 | 122574 | 0,29 | -5,05 | 107 | 1.00:1.23:0.03 |
| Q8CGK3 | Lon protease homolog mitochondrial | - | 1 | 105843 | -0,33 | -2,96 | 19,8 | 1.00:0.80:0.13 |
| A0A0J9YVJ0 | Maltase-glucoamylase (Fragment) | - | 3 | 112822 | -2,66 | -6,79 | 78,99 | 1.00:0.16:0.01 |
| Q9D3H2 | Odorant-binding protein 1a | - | 39 | 18469 | 0,54 | -4,21 | 147,5 | 1.00:1.44:0.05 |
| A2AEN9 | Predicted gene 5938 | - | 42 | 18508 | 0,46 | -5,11 | 130,41 | 1.00:1.37:0.03 |
| A0A2R8VHP3 | Predicted pseudogene 5478 | - | 11 | 57920 | 2,49 | 2,50 | 26,63 | 1.00:5.62:5.64 |
| Q9WVF5 | Receptor protein-tyrosine kinase | - | 7 | 72907 | 1,04 | 2,05 | 32,28 | 1.00:2.07:4.14 |
| Q6UGQ3 | Secretoglobin family 2B member 2 | - | 35 | 12857 | 0,04 | -6,40 | 132,27 | 1.00:1.03:0.01 |

*Significance set to ≥ 13 meaning p value ≤ 0,05 (=-Log_10_(pvalue)*10)*

**Supplementary Table 3.** List of differentially abundant proteins shown on Figure 2B, C and D.

| **Proteins shown on Fig. 2B** | | |  |  |  |  |  |  |
| --- | --- | --- | --- | --- | --- | --- | --- | --- |
| **Accession** | **Description** | **Coverage (%)** | | **#Peptides** | **Avg. Mass** | **Control Area** | **5th week post-infection Area** | **7th week post-infection Area** |
| Q60994 | Adiponectin | | 13 | 3 | 26809 | 4,17E+07 | 1,84E+07 | 1,02E+07 |
| P09813 | Apolipoprotein A-II | | 15 | 2 | 11309 | 7,44E+08 | 7,73E+08 | 3,50E+08 |
| Q05020 | Apolipoprotein C-II | | 33 | 3 | 10741 | 1,30E+07 | 4,87E+05 | 1,36E+07 |
| P33622 | Apolipoprotein C-III | | 46 | 3 | 10982 | 2,65E+08 | 1,30E+08 | 5,27E+08 |
| Q07968 | Coagulation factor XIII B chain | | 5 | 3 | 76195 | 5,37E+06 | 4,67E+06 | 1,64E+06 |
| A2A998 | Complement component C8 alpha chain | | 9 | 4 | 61008 | 6,08E+06 | 1,08E+07 | 2,53E+06 |
| Q06770 | Corticosteroid-binding globulin | | 28 | 12 | 44769 | 2,53E+08 | 2,49E+08 | 6,77E+07 |
| P01942 | Hemoglobin subunit alpha | | 61 | 8 | 15085 | 1,41E+09 | 6,89E+08 | 2,85E+08 |
| P02088 | Hemoglobin subunit beta-1 | | 84 | 5 | 15840 | 6,58E+08 | 4,27E+08 | 1,74E+08 |
| P02089 | Hemoglobin subunit beta-2 | | 80 | 5 | 15878 | 1,98E+08 | 1,04E+08 | 4,33E+07 |
| P70389 | Insulin-like growth factor-binding protein complex acid labile subunit | | 11 | 5 | 66960 | 7,25E+06 | 8,73E+06 | 2,71E+06 |
| Q61730 | Interleukin-1 receptor accessory protein | | 13 | 6 | 65741 | 2,16E+07 | 2,30E+07 | 5,54E+06 |
| Q922U2 | Keratin type II cytoskeletal 5 | | 21 | 6 | 61767 | 1,91E+07 | 6,31E+06 | 6,70E+06 |
| P50446 | Keratin type II cytoskeletal 6A | | 27 | 4 | 59335 | 4,39E+07 | 2,61E+06 | 3,84E+06 |
| Q8VED5 | Keratin type II cytoskeletal 79 | | 12 | 2 | 57552 | 5,48E+07 | 1,55E+07 | 3,16E+07 |
| P42703 | Leukemia inhibitory factor receptor | | 12 | 11 | 122574 | 1,39E+07 | 1,70E+07 | 4,21E+05 |
| Q8CGK3 | Lon protease homolog mitochondrial | | 1 | 1 | 105843 | 5,24E+06 | 4,17E+06 | 6,73E+05 |
| A0A0J9YVJ0 | Maltase-glucoamylase (Fragment) | | 3 | 3 | 112822 | 3,85E+06 | 6,10E+05 | 3,48E+04 |
| Q9D3H2 | Odorant-binding protein 1a | | 39 | 4 | 18469 | 9,52E+07 | 1,38E+08 | 5,14E+06 |
| O35930 | Platelet glycoprotein Ib alpha chain | | 4 | 2 | 80055 | 2,36E+06 | 9,29E+05 | 4,49E+05 |
| A2AEN9 | Predicted gene 5938 | | 42 | 4 | 18508 | 3,56E+07 | 4,89E+07 | 1,03E+06 |
| Q6UGQ3 | Secretoglobin family 2B member 2 | | 35 | 3 | 12857 | 3,15E+07 | 3,24E+07 | 3,72E+05 |
| **Proteins shown on Fig. 2C** | | |  |  |  |  |  |  |
| **Accession** | **Description** | | **Coverage (%)** | **#Peptides** | **Avg. Mass** | **Control Area** | **5th week post-infection Area** | **7th week post-infection Area** |
| P29699 | Alpha-2-HS-glycoprotein | | 53 | 16 | 37326 | 2,05E+09 | 1,73E+09 | 4,93E+09 |
| Q3UTR7 | Angiotensin 1-10 | | 14 | 6 | 52670 | 6,61E+06 | 7,39E+06 | 1,53E+07 |
| P06728 | Apolipoprotein A-IV | | 84 | 36 | 45029 | 3,56E+08 | 2,54E+08 | 1,04E+09 |
| E9Q1Y3 | Apolipoprotein B-100 (Fragment) | | 10 | 36 | 503910 | 6,29E+06 | 7,99E+06 | 3,14E+07 |
| Q61268 | Apolipoprotein C-IV | | 16 | 2 | 14288 | 1,21E+07 | 7,42E+06 | 2,62E+07 |
| P51910 | Apolipoprotein D | | 31 | 5 | 21530 | 3,67E+07 | 3,17E+07 | 9,34E+07 |
| P01027 | Complement C3 | | 70 | 107 | 186483 | 5,39E+08 | 4,08E+08 | 1,77E+09 |
| D3YXF5 | Complement component 7 | | 6 | 4 | 93338 | 4,46E+05 | 1,95E+05 | 5,67E+06 |
| A0A0A6YVP8 | Complement factor H | | 59 | 23 | 80361 | 9,69E+07 | 1,21E+08 | 2,51E+08 |
| P14847 | C-reactive protein | | 10 | 2 | 25360 | 1,73E+06 | 2,17E+06 | 5,03E+06 |
| E9PV24 | Fibrinogen alpha chain | | 44 | 35 | 87429 | 1,18E+09 | 1,35E+09 | 3,57E+09 |
| Q8K0E8 | Fibrinogen beta chain | | 77 | 35 | 54753 | 9,86E+08 | 1,11E+09 | 2,93E+09 |
| Q8VCM7 | Fibrinogen gamma chain | | 73 | 32 | 49391 | 1,03E+09 | 1,31E+09 | 2,48E+09 |
| P01878 | Ig alpha chain C region | | 42 | 8 | 36876 | 1,14E+08 | 5,71E+07 | 4,68E+08 |
| P01867 | Ig gamma-2B chain C region | | 35 | 10 | 44259 | 5,47E+07 | 4,82E+07 | 1,18E+08 |
| A0A075B5P5 | Immunoglobulin heavy constant gamma 3 (Fragment) | | 51 | 11 | 36317 | 1,55E+08 | 1,83E+08 | 3,83E+08 |
| A0A075B5R6 | Immunoglobulin heavy variable 11-1 (Fragment) | | 28 | 2 | 13208 | 1,87E+06 | 2,12E+06 | 1,45E+07 |
| A0A140T8P3 | Immunoglobulin kappa chain variable 15-103 (Fragment) | | 14 | 2 | 12547 | 1,64E+06 | 3,61E+05 | 1,00E+07 |
| Q61704 | Inter-alpha-trypsin inhibitor heavy chain H3 | | 24 | 18 | 99358 | 3,99E+07 | 4,81E+07 | 1,66E+08 |
| P11680 | Properdin | | 14 | 5 | 50327 | 1,15E+07 | 8,59E+06 | 4,34E+07 |
| Q07456 | Protein AMBP | | 22 | 6 | 39029 | 1,75E+08 | 1,46E+08 | 3,93E+08 |
| Q921I1 | Serotransferrin | | 74 | 55 | 76724 | 5,17E+09 | 5,93E+09 | 1,17E+10 |
| **Proteins shown on Fig. 2D** | | |  |  |  |  |  |  |
| **Accession** | **Description** | | **Coverage (%)** | **#Peptides** | **Avg. Mass** | **Control Area** | **5th week post-infection Area** | **7th week post-infection Area** |
| Q00897 | Alpha-1-antitrypsin 1-4 | | 45 | 3 | 45998 | 1,39E+07 | 6,17E+07 | 4,53E+08 |
| Q9QWK4 | CD5 antigen-like | | 42 | 14 | 38863 | 2,84E+07 | 6,38E+07 | 1,25E+08 |
| P06683 | Complement component C9 | | 10 | 6 | 62002 | 1,61E+06 | 9,96E+06 | 9,16E+06 |
| Q91X72 | Hemopexin | | 60 | 28 | 51318 | 1,44E+09 | 3,19E+09 | 4,06E+09 |
| Q8HWB2 | Histocompatibility 2 Q region locus 4 | | 15 | 5 | 39618 | 4,73E+05 | 1,15E+06 | 4,77E+06 |
| A0A075B5P4 | Ig gamma-1 chain C region secreted form (Fragment) | | 52 | 11 | 35752 | 8,15E+07 | 1,57E+09 | 2,44E+09 |
| P01868 | Ig gamma-1 chain C region secreted form | | 56 | 13 | 35705 | 6,78E+05 | 4,36E+07 | 1,07E+08 |
| P01806 | Ig heavy chain V region 441 | | 72 | 8 | 12911 | 2,74E+07 | 6,61E+07 | 1,13E+09 |
| P01790 | Ig heavy chain V region M511 | | 52 | 5 | 13652 | 7,09E+06 | 1,63E+07 | 4,35E+07 |
| P01801 | Ig heavy chain V-III region J606 | | 43 | 4 | 12810 | 5,57E+06 | 1,39E+07 | 5,86E+07 |
| P03976 | Ig kappa chain V-II region 17S29.1 | | 27 | 3 | 12390 | 3,99E+06 | 4,96E+07 | 8,73E+08 |
| P01629 | Ig kappa chain V-II region 2S1.3 | | 45 | 4 | 12221 | 4,71E+05 | 1,00E+06 | 3,15E+07 |
| P01630 | Ig kappa chain V-II region 7S34.1 | | 27 | 3 | 12496 | 8,84E+05 | 2,51E+06 | 1,03E+07 |
| P01662 | Ig kappa chain V-III region ABPC 22/PC 9245 | | 65 | 5 | 12041 | 3,12E+05 | 1,62E+07 | 4,57E+07 |
| P01656 | Ig kappa chain V-III region MPC70 | | 46 | 5 | 11904 | 1,31E+06 | 1,33E+07 | 4,97E+06 |
| P01668 | Ig kappa chain V-III region PC 7210 | | 36 | 4 | 11950 | 5,10E+06 | 2,78E+07 | 4,34E+07 |
| P01644 | Ig kappa chain V-V region HP R16.7 | | 63 | 6 | 11910 | 2,59E+07 | 1,21E+08 | 2,93E+08 |
| P01635 | Ig kappa chain V-V region K2 (Fragment) | | 45 | 5 | 12581 | 6,29E+05 | 1,45E+07 | 3,94E+07 |
| P01638 | Ig kappa chain V-V region L6 (Fragment) | | 28 | 3 | 12986 | 1,82E+06 | 4,10E+07 | 3,66E+07 |
| P01642 | Ig kappa chain V-V region L7 (Fragment) | | 29 | 3 | 12615 | 3,81E+06 | 4,09E+07 | 5,31E+07 |
| P01843 | Ig lambda-1 chain C region | | 81 | 5 | 11575 | 2,97E+07 | 6,56E+07 | 2,79E+08 |
| P01723 | Ig lambda-1 chain V region | | 56 | 3 | 12222 | 8,81E+06 | 2,60E+07 | 6,86E+07 |
| P01844 | Ig lambda-2 chain C region | | 71 | 4 | 11255 | 7,70E+06 | 5,33E+07 | 1,06E+08 |
| A0A075B5Q9 | Immunoglobulin heavy variable 5-15 | | 33 | 3 | 12943 | 1,44E+05 | 4,07E+06 | 3,95E+06 |
| A0A075B5S9 | Immunoglobulin heavy variable 9-4 | | 31 | 3 | 10934 | 7,84E+06 | 6,58E+07 | 6,17E+07 |
| A0A075B5T5 | Immunoglobulin heavy variable V1-5 | | 19 | 1 | 10846 | 9,76E+06 | 1,04E+08 | 9,81E+07 |
| A0A075B5X6 | Immunoglobulin heavy variable V1-67 | | 11 | 1 | 10976 | 1,28E+06 | 1,02E+07 | 1,38E+07 |
| A0A075B5X4 | Immunoglobulin heavy variable V8-11 (Fragment) | | 22 | 2 | 13082 | 1,51E+06 | 2,31E+07 | 2,75E+07 |
| A0A075B6D5 | Immunoglobulin kappa chain variable 19-93 | | 19 | 2 | 12735 | 2,06E+06 | 2,76E+07 | 7,39E+07 |
| A0A140T8P5 | Immunoglobulin kappa chain variable 8-24 (Fragment) | | 35 | 4 | 13264 | 3,22E+06 | 7,92E+06 | 2,45E+07 |
| A0A140T8M3 | Immunoglobulin kappa chain variable 8-30 (Fragment) | | 35 | 4 | 13335 | 1,98E+06 | 1,01E+07 | 1,89E+07 |
| A0A140T8N0 | Immunoglobulin kappa chain variable 9-120 (Fragment) | | 51 | 5 | 12805 | 6,92E+06 | 1,71E+07 | 6,16E+07 |
| P01837 | Immunoglobulin kappa constant | | 87 | 9 | 11934 | 9,90E+08 | 3,50E+09 | 8,51E+09 |
| A0A0B4J1H7 | Immunoglobulin kappa variable 1-135 (Fragment) | | 46 | 7 | 13305 | 1,62E+07 | 2,77E+08 | 1,92E+08 |
| A0A140T8P6 | Immunoglobulin kappa variable 12-46 (Fragment) | | 45 | 4 | 12562 | 3,85E+05 | 3,13E+07 | 4,04E+07 |
| A0A0B4J1I1 | Immunoglobulin kappa variable 16-104 (Fragment) | | 23 | 2 | 12875 | 6,12E+06 | 5,38E+07 | 8,38E+07 |
| A0A075B5J9 | Immunoglobulin kappa variable 17-127 | | 14 | 1 | 12330 | 2,07E+06 | 3,21E+07 | 3,86E+07 |
| A0A075B5N9 | Immunoglobulin kappa variable 3-7 | | 36 | 3 | 10856 | 3,43E+06 | 2,51E+07 | 3,05E+07 |
| A0A0B4J1I9 | Immunoglobulin kappa variable 4-55 (Fragment) | | 43 | 3 | 12736 | 4,73E+06 | 3,09E+07 | 2,90E+07 |
| A0A0B4J1I7 | Immunoglobulin kappa variable 4-68 (Fragment) | | 17 | 3 | 12752 | 5,16E+05 | 9,12E+06 | 1,48E+07 |
| A0A075B5M7 | Immunoglobulin kappa variable 5-39 | | 47 | 3 | 10345 | 1,88E+06 | 1,05E+07 | 3,70E+07 |
| A0A140T8M5 | Immunoglobulin kappa variable 6-15 (Fragment) | | 53 | 6 | 12757 | 2,30E+06 | 4,21E+07 | 6,95E+07 |
| A0A140T8Q3 | Immunoglobulin kappa variable 6-17 (Fragment) | | 45 | 6 | 12760 | 1,65E+06 | 1,47E+07 | 1,86E+07 |
| A0A140T8N9 | Immunoglobulin kappa variable 6-32 (Fragment) | | 45 | 5 | 12656 | 3,82E+06 | 3,77E+07 | 1,08E+08 |
| A0A075B5N3 | Immunoglobulin kappa variable 8-28 | | 42 | 4 | 10903 | 6,54E+05 | 2,46E+07 | 7,75E+06 |
| A0A075B664 | Immunoglobulin lambda variable 2 | | 14 | 2 | 12165 | 2,74E+05 | 1,28E+06 | 4,73E+06 |
| Q6S9I0 | Kininogen 2 | | 30 | 2 | 47887 | 2,21E+06 | 7,33E+06 | 1,17E+07 |
| P09581 | Macrophage colony-stimulating factor 1 receptor | | 5 | 4 | 109179 | 3,77E+06 | 1,02E+07 | 9,63E+06 |
| Q60963 | Platelet-activating factor acetylhydrolase | | 10 | 4 | 49258 | 5,34E+05 | 1,15E+06 | 5,50E+06 |
| A0A2R8VHP3 | Predicted pseudogene 5478 | | 11 | 1 | 57920 | 7,02E+05 | 3,94E+06 | 3,96E+06 |
| Q9WVF5 | Receptor protein-tyrosine kinase | | 7 | 4 | 72907 | 2,21E+06 | 4,56E+06 | 9,15E+06 |
| Q8BND5 | Sulfhydryl oxidase 1 | | 25 | 17 | 82785 | 7,65E+06 | 1,89E+07 | 2,20E+07 |

**Supplementary Table 4.** Uniquely identified proteins on the quantitative analysis.

| **Accession** | **Description** | **Coverage (%)** | **Peptides** | **Molecular Mass (Da)** | **Control Sample Area** | **5^th^ week post-infection Area** | **7^th^ week post-infection Area** |
| --- | --- | --- | --- | --- | --- | --- | --- |
| Q19LI2 | Alpha-1B-glycoprotein | 9 | 5 | 56554 | 2,62E+07 | 3,63E+06 | 0 |
| P00920 | Carbonic anhydrase 2 | 7 | 2 | 29033 | 9,34E+05 | 3,97E+05 | 0 |
| D3Z6T3 | Cathepsin E | 5 | 2 | 39247 | 0 | 0 | 1,72E+09 |
| Q61147 | Ceruloplasmin | 49 | 1 | 121151 | 2,64E+06 | 3,19E+06 | 0 |
| Q91X70 | Complement component 6 | 3 | 2 | 86631 | 0 | 0 | 9,04E+06 |
| Q61646 | Haptoglobin | 50 | 20 | 38752 | 0 | 8,22E+07 | 1,24E+07 |
| Q9R098 | Hepatocyte growth factor activator | 7 | 3 | 70568 | 4,53E+06 | 5,78E+06 | 0 |
| P06336 | Ig epsilon chain C region | 32 | 9 | 47321 | 0 | 3,34E+06 | 1,23E+07 |
| P03977 | Ig kappa chain V-III region 50S10.1 | 46 | 5 | 12042 | 0 | 7,51E+06 | 1,01E+07 |
| A0A075B5R4 | Immunoglobulin heavy variable 14-1 (Fragment) | 26 | 3 | 12992 | 0 | 0 | 1,05E+07 |
| A0A075B5Q3 | Immunoglobulin heavy variable 2-5 | 20 | 2 | 12557 | 0 | 1,55E+07 | 2,38E+06 |
| A0A075B5R3 | Immunoglobulin heavy variable 7-2 | 20 | 2 | 13351 | 0 | 1,57E+06 | 5,56E+07 |
| A0A075B5R2 | Immunoglobulin heavy variable 7-3 (Fragment) | 28 | 3 | 13457 | 0 | 7,54E+06 | 6,02E+06 |
| A0A075B5R9 | Immunoglobulin heavy variable V14-3 (Fragment) | 27 | 3 | 12975 | 0 | 1,26E+07 | 1,11E+07 |
| A0A075B5Y1 | Immunoglobulin heavy variable V1-74 | 29 | 3 | 12900 | 0 | 1,22E+06 | 2,37E+08 |
| A0A0G2JDE1 | Immunoglobulin heavy variable V8-12 (Fragment) | 13 | 1 | 13263 | 0 | 2,33E+07 | 5,22E+07 |
| A0A075B5M8 | Immunoglobulin kappa chain variable 12-38 | 34 | 3 | 12428 | 0 | 2,04E+06 | 1,94E+07 |
| A0A075B5K7 | Immunoglobulin kappa chain variable 14-100 | 14 | 1 | 12650 | 0 | 5,71E+06 | 8,84E+06 |
| A0A140T8M0 | Immunoglobulin kappa variable 1-117 (Fragment) | 43 | 4 | 13117 | 0 | 8,64E+07 | 0 |
| A0A140T8M2 | Immunoglobulin kappa variable 12-44 (Fragment) | 45 | 5 | 12557 | 0 | 4,44E+07 | 7,84E+06 |
| A0A075B5K0 | Immunoglobulin kappa variable 14-126 (Fragment) | 19 | 2 | 13058 | 6,10E+05 | 0 | 0 |
| A0A140T8P1 | Immunoglobulin kappa variable 6-14 (Fragment) | 48 | 6 | 12848 | 0 | 1,04E+06 | 3,75E+07 |
| Q9Z2K1 | Keratin type I cytoskeletal 16 | 13 | 2 | 51606 | 2,54E+06 | 0 | 0 |
| Q9QWL7 | Keratin type I cytoskeletal 17 | 40 | 7 | 48162 | 2,92E+07 | 0 | 0 |
| P19001 | Keratin type I cytoskeletal 19 | 13 | 2 | 44542 | 4,60E+06 | 0 | 0 |
| B8JI96 | Major urinary protein 14 | 23 | 3 | 17365 | 5,60E+06 | 1,81E+06 | 0 |
| P11034 | Mast cell protease 1 | 21 | 4 | 27013 | 0 | 0 | 8,84E+06 |
| A2AEP0 | Odorant-binding protein 1b | 53 | 9 | 19394 | 2,31E+07 | 6,10E+07 | 0 |
| A0A140LIG1 | Phosphatidylinositol N-acetylglucosaminyltransferase subunit A | 3 | 1 | 27766 | 8,13E+06 | 3,48E+05 | 0 |
| Q9Z126 | Platelet factor 4 | 20 | 2 | 11243 | 2,16E+06 | 0 | 0 |
| O70570 | Polymeric immunoglobulin receptor | 4 | 3 | 84999 | 0 | 0 | 7,84E+06 |

**Supplementary Table 5.** Serum lipid profile of experimental groups. Results are expressed as mean ± standard deviation. The letters indicate significant difference between groups (a = p<0,05; b = p<0,001).

|  | Group | | |  |
| --- | --- | --- | --- | --- |
|  | 5^th^ week post infection | | 7^th^ week post infection | |
| Lipid’s parameters | Control | Infected | Control | Infected |
| Total Cholesterol (mg/dL) | 67,36 ± 3,376^a^ | 60,93 ± 6,973^a^ | 63,37 ± 5,279 | 63,06 ± 7,871 |
| HDL Cholesterol (mg/dL) | 38,58 ± 2,754^b^ | 31,71 ± 5,349^b^ | 35,82 ± 6,3 | 31,6 ± 15,24 |
| Atherogenic fraction (mg/dL) | 28,87 ± 4,157 | 29,24 ± 4,822 | 29,68 ± 3,522^a^ | 39,93 ± 6,689^a^ |
| VLDL Cholesterol (mg/dL) | 25,15 ± 6,909^b^ | 11,13 ± 1,97^b^ | 16,25 ± 3,213^a^ | 19,27 ± 5,491^a^ |
| Triglycerides (mg/dL) | 125,8 ± 34,55^b^ | 55,67 ± 9,852^b^ | 81,25 ± 16,06^a^ | 96,33± 27,46^a^ |
